# Polyglutamine-expanded ATXN7 alters a specific epigenetic signature underlying photoreceptor identity gene expression in SCA7 mouse retinopathy

**DOI:** 10.1186/s12929-022-00892-1

**Published:** 2022-12-20

**Authors:** Anna Niewiadomska-Cimicka, Antoine Hache, Stéphanie Le Gras, Céline Keime, Tao Ye, Aurelie Eisenmann, Imen Harichane, Michel J. Roux, Nadia Messaddeq, Emmanuelle Clérin, Thierry Léveillard, Yvon Trottier

**Affiliations:** 1 Institute of Genetics and Molecular and Cellular Biology (IGBMC), INSERM U1258, CNRS UMR7104, University of Strasbourg, 67404 Illkirch, France; 2 Department of Genetics, INSERM, CNRS, Institut de la Vision, Sorbonne University, 75012 Paris, France

**Keywords:** Spinocerebellar ataxia type 7, Photoreceptor dystrophy, SAGA, Epigenomics, Transcriptomics, Neuronal identity

## Abstract

**Background:**

Spinocerebellar ataxia type 7 (SCA7) is a neurodegenerative disorder that primarily affects the cerebellum and retina. SCA7 is caused by a polyglutamine expansion in the ATXN7 protein, a subunit of the transcriptional coactivator SAGA that acetylates histone H3 to deposit narrow H3K9ac mark at DNA regulatory elements of active genes. Defective histone acetylation has been presented as a possible cause for gene deregulation in SCA7 mouse models. However, the topography of acetylation defects at the whole genome level and its relationship to changes in gene expression remain to be determined.

**Methods:**

We performed deep RNA-sequencing and chromatin immunoprecipitation coupled to high-throughput sequencing to examine the genome-wide correlation between gene deregulation and alteration of the active transcription marks, *e.g.* SAGA-related H3K9ac, CBP-related H3K27ac and RNA polymerase II (RNAPII), in a SCA7 mouse retinopathy model.

**Results:**

Our analyses revealed that active transcription marks are reduced at most gene promoters in SCA7 retina, while a limited number of genes show changes in expression. We found that SCA7 retinopathy is caused by preferential downregulation of hundreds of highly expressed genes that define morphological and physiological identities of mature photoreceptors. We further uncovered that these photoreceptor genes harbor unusually broad H3K9ac profiles spanning the entire gene bodies and have a low RNAPII pausing. This broad H3K9ac signature co-occurs with other features that delineate superenhancers, including broad H3K27ac, binding sites for photoreceptor specific transcription factors and expression of enhancer-related non-coding RNAs (eRNAs). In SCA7 retina, downregulated photoreceptor genes show decreased H3K9 and H3K27 acetylation and eRNA expression as well as increased RNAPII pausing, suggesting that superenhancer-related features are altered.

**Conclusions:**

Our study thus provides evidence that distinctive epigenetic configurations underlying high expression of cell-type specific genes are preferentially impaired in SCA7, resulting in a defect in the maintenance of identity features of mature photoreceptors. Our results also suggest that continuous SAGA-driven acetylation plays a role in preserving post-mitotic neuronal identity.

**Supplementary Information:**

The online version contains supplementary material available at 10.1186/s12929-022-00892-1.

## Background

Spinocerebellar ataxia type 7 (SCA7) is an autosomal dominant neurodegenerative disorder characterized by loss of balance and fine movements, poor coordination, language problems as well as central vision deficit, which can evolve toward complete blindness [1]. SCA7 is caused by the expansion of a CAG repeat encoding a polyglutamine (polyQ) tract in ATAXIN-7 (ATXN7) [2, 3], and hence belongs to the family of 9 polyQ neurodegenerative disorders, which include 5 other SCAs (SCA1, SCA2, SCA3, SCA6 and SCA17) and Huntington’s disease (HD). ATXN7 is a ubiquitously expressed protein and a core component of the multiprotein complex Spt-Ada-Gcn5 Acetyltransferase (SAGA), which owns histone modification activities to establish specific epigenetic patterns for RNA polymerase II (RNAPII) transcription [4]. In particular, SAGA harbors either GCN5 (KAT2A) or PCAF (KAT2B) acetyl-transferase activity to acetylate lysine 9 of histone H3 (H3K9ac). H3K9ac is a typical epigenetic mark of transcriptionally active gene promoters and distal regulatory regions and is often associated with H3K27ac [5]. The co-activation function of SAGA has been mainly reported in yeast, cultured cells and fly, but is still poorly characterized in mammalian tissues and pathological situations [6].

SCA7 has a unique feature among polyQ diseases to cause retina degeneration. Early studies of SCA7 mice have shown that dysfunction of retinal photoreceptors occurs in the absence of substantial cell death and correlates with the decreased expression of phototransduction genes [7–11]. To capture light, photoreceptors bear a specialized ciliary structure called the outer segments (OS) that contain molecular components for phototransduction [12]. OS are an identity feature of mature photoreceptors and require partial daily renewal to ensure the maintenance of photosensitivity. In SCA7 mice, photoreceptor OS progressively shorten over time, suggesting a deficit in OS renewal [7–11]. Interestingly, in zebrafish, depletion of *atxn7* during retinal development impairs the formation of photoreceptor OS [13, 14]. Together, this suggested that polyQ expansion in ATXN7 alters specific mechanisms involved in OS maintenance of mature photoreceptors in SCA7.

OS formation and maintenance are under the control of cell-type specific transcription factors, such as the cone-rod homeobox protein (CRX) and the neural retina leucine zipper protein (NRL) [15, 16]. Mutated polyQ-containing ATXN7 (mATXN7) interferes with CRX and NRL transactivation activities in vitro reporter gene assays [7, 17]. However, the mechanism underlying mATXN7 interferences remains unclear. While mATXN7 interacts with CRX in vitro (but not with NRL) [7], the direct mATXN7-CRX interaction was not detected in retina extracts [18]. mATXN7 can incorporate into SAGA complex [18] and reduces its histone acetylation activity (HAT) in vitro [19, 20]. However, in SCA7 mouse retina, promoters of CRX regulated genes were found either hypo- or hyperacetylated on histone H3, leading to inconsistent results [18, 19]. Since analyses were carried out on a limited number of gene promoters and using different transgenic mice, it is unclear whether any of these alterations parallel those occurring in the retina of patients. Therefore, the mechanisms underlying SCA7 retinopathy need to be clarified by a global investigation of the effect of mATXN7 on SAGA-related epigenetic marks and co-activation functions.

Increasing evidence indicates that epigenomic alterations are implicated in neurodegenerative disorders and could lead to deficit in transcriptional programs involved in the maintenance of neuron maturation [21]. For instances, studies in HD and Alzheimer’s disease reported correlations between transcriptional deregulation and changes in histone acetylation at cell-type specific enhancers [22–24]. Enhancers play key roles in gene expression as they are bound by specific regulatory factors, interact with gene promoters within genomic topologically associated domains (TADs) by looping out chromatin, and regulate RNAPII recruitment and dynamics [25]. In addition, a subset of enhancers is transcribed into long non-coding RNAs (lncRNAs), the so-called eRNAs, that may also facilitate enhancer-promoter interactions and RNAPII dynamics [26, 27]. In particular, clusters of enhancers, called superenhancers, are spread over large genomic regions and drive the expression of genes that define cell identity and cell-type specific functions [28]. A recent study showed that the H3K27 acetyl-transferases CBP (KAT3A) and P300 (KAT3B) jointly play a critical role in the maintenance of superenhancers underlying neuronal identity [29]. CBP HAT function is thought to be altered in polyQ disorders, as a result of its sequestration in intranuclear polyQ aggregates formed by the mutant polyQ proteins [30]. It was suggested that SAGA, which is recruited at enhancers, may also participate to cell-specific transcription programs [31]*.* Whether deficits in histone acetylation at enhancers or superenhancers contribute to gene deregulation in SCA7 remain to be demonstrated.

Here, we have combined RNA sequencing (RNA-seq) and chromatin immunoprecipitation coupled to high-throughput sequencing (ChIP-seq) to perform the first genome-wide correlative study between gene deregulation and alteration of histone acetylation in the retina of SCA7 knock-in mice. Our results first reveal that promoters of most expressed genes in SCA7 retina present an important decrease of H3K9 acetylation, which correlates with reduced levels of other active transcription marks such as H3K27 acetylation and RNAPII occupancy, However, this local epigenetic alteration does not lead to a global change in gene expression. We further uncovered that unusually broad profiles of H3K9 acetylation encompass the entire loci of a subset of genes that are highly expressed in wild-type (WT) retina, present low levels of paused RNAPII and are involved in OS morphology and physiological identities of mature photoreceptors. This distinctive H3K9ac signature coincides with other features which typically define superenhancer regions, such as broad H3K27 acetylation, enrichment in binding motifs for CRX and NRL and expression of putative eRNAs. In SCA7 retina, alteration of the broad H3K9 and H3K27 acetylation and eRNA expression correlates with the predominant downregulation of these photoreceptor identity genes, suggesting that superenhancer functions supporting the high expression of these genes are impaired. Our findings thus provide evidence that critical epigenetic mechanisms involved in the maintaining specific morphology, function and gene expression of mature photoreceptors are impaired in SCA7 mouse pathology.

## Methods

### Mouse information

The SCA7^140Q/5Q^ and SCA7^140Q/140Q^ knock-in mouse lines harbor expansion ranging from 140 to 150 CAG repeats in exon1 of the *Atxn7* locus [11]. Mice were kept on C57Bl/6J background and breed in the Mouse Clinical Institute (Illkirch, France). All animal procedures were carried out in strict accordance with the French national laws for laboratory animal welfare and the guidelines of the Federation of European Laboratory Animal Science Associations, based on European Union Legislation (Directive 2010/63/EU). Experiments were prior approved by the local Ethics Committees under the supervision of the Ministry of Higher Education, Research and Innovation in France (agreement numbers: #5149-20160422171329v1, 14199-2018032117483097v2 and #13869-2018021216211243v5). Mice were housed in a 12 h light/dark cycle with free access to food and water. Genotyping was performed by PCR according to protocols previously described [8]. All of WT, SCA7^140Q/5Q^ and SCA7^140Q/140Q^ males and females were analyzed.

### Photoreceptor-enriched samples

Photoreceptor outer and inner layers were sectioned from WT retina of 2 months old mice by vibratome sectioning [32]. Briefly, the dissected retina was flat-embedded in 4% gelatin on top of a basement of 20% gelatin (gelatin from porcine skin type A, Sigma G2500) in CO_2_-independent medium without l-glutamine and 20 µM of gentamicin, with the inner retina facing up. Using a vibratome (Leica) and double-edged razor blade, the 100 µm corresponding to inner retina and an intermediary section of 30 µm were sectioned and discarded. Next, the 200 µm thick section of the photoreceptor layer was recovered. Gelatin was removed by heating the outer layer 5 min at 37 °C and the photoreceptor layer was collected and put in liquid nitrogen.

### Quantitative (RT)-PCR

*mRNA Reverse Transcription (RT).* Total RNAs (250 ng) were treated with 2 units of DNAse1 (Roche, 4716728001) for 22 min at 37 °C, followed by thermic inactivation for 20 min at 70 °C. Samples were then reverse transcribed using a mix of oligo-dT and random hexamer primers with the SuperScript IV kit (ThermoFisher Scientific), following the manufacturer’s instructions.

*Targeted tagged RT for putative eRNA and mRNA expression from photoreceptor gene loci*. Total RNAs (1000 ng for putative eRNA/ 250 ng for mRNA) were treated with DNAse1 and reverse transcribed using SuperScript IV kit as described above. The strand specificity for qPCR was guaranteed by using tagged primers during the RT, as published in Craggs et al. [33] (Additional file 1: Table S1). These primers contain a 19–21 unique nucleotide ‘tag’ sequences unrelated to *Mus musculus* genome added to their 5′ end.

*qPCR.* Real-time qPCR reactions were performed on cDNAs (1/20 dilution for mRNA, 1/5 for putative eRNA) or ChIP samples (1/2 dilution) using QuantiTect SYBR Green PCR Master Mix (Qiagen), and gene and/or tag specific primers (Additional file 1: Table S1). Gene expression was normalized against *Hprt or 36B4 (Rplp0)*.

### RNA-seq

Total RNA samples were extracted from 2 retinas/animal/sample (4 WT and 3 SCA7^140Q/140Q^ knock-in mice) using RNeasy Micro Kit (Qiagen) following the manufacturer’s instruction. Total RNA-Seq libraries were generated from 1 µg of total RNA using TruSeq Stranded Total RNA Library Prep Gold kit and TruSeq RNA Single Indexes kits A and B (Illumina, San Diego, CA), according to manufacturer's instructions. Briefly, cytoplasmic and mitochondrial ribosomal RNA (rRNA) were removed using biotinylated, target-specific oligos combined with Ribo-Zero rRNA removal beads. Following purification, the remaining RNA was fragmented into small pieces using divalent cations at 94 °C for 2 min. RNA fragments were then copied into first strand cDNA using reverse transcriptase and random primers followed by second strand cDNA synthesis using DNA Polymerase I and RNase H. Strand specificity was achieved by replacing dTTP with dUTP during second strand synthesis. The double stranded cDNA fragments were blunted using T4 DNA polymerase, Klenow DNA polymerase and T4 PNK. A single ‘A’ nucleotide was added to the 3' ends of the blunt DNA fragments using a Klenow fragment (3′–5′ exo minus) enzyme. The cDNA fragments were ligated to double stranded adapters using T4 DNA Ligase. The ligated products were enriched by PCR amplification (30 s at 98 °C; [10 s at 98 °C, 30 s at 60 °C, 30 s at 72 °C] × 12 cycles; 5 min at 72 °C). Surplus PCR primers were removed using AMPure XP beads (Beckman-Coulter, Villepinte, France) and the final cDNA libraries were quality controlled and quantified using capillary electrophoresis.

Libraries were sequenced on Illumina Hiseq 4000 sequencer as single-read 50 bases following Illumina’s instructions. Image analysis and base calling were performed using RTA 2.7.3 and bcl2fastq. Reads were mapped onto mm10 assembly of mouse genome using Tophat v2.0.14 with bowtie2 v2.1.0 aligner [34, 35]. Quantification of gene expression was performed using HTSeq v0.6.1 and gene annotations from Ensembl release 90 [36]. Comparison between WT and SCA7 samples was performed using DESeq2 Bioconductor library (DESeq2 v1.16.1). p-values were adjusted for multiple testing using the Benjamini and Hochberg method [37]. Gene expression values (indicated as reads per kilobase (RPK) throughout the result section) correspond to read counts normalized across libraries with the method proposed by Anders and Huber [38] (size factors computed using DESeq2 v1.16.1 Bioconductor library) and divided by gene length (calculated as the median of the length of all transcripts corresponding to this gene in kb). Genes were considered as expressed in the retina only when RPK ≥ 1 in all WT samples.

### Chromatin immunoprecipitation (ChIP) and sequencing

Chromatin was prepared from 6 retinas. Following cross-linking in 1% paraformaldehyde (PFA) at room temperature for 12 min, glycine was added to a final concentration of 0.46 M and incubated for 10 min at room temperature. Samples were washed in cold PBS/PIC and homogenized in lysis buffer (50 mM Hepes pH 7.5, 1 mM EDTA, 1% Triton X-100, 0.1% Na-deoxycholate, 1% SDS, 1 × PIC). Chromatin was sonicated for 10 min using a Covaris and centrifuged at 13 000 rpm for 5 min. Each 100 μl of chromatin sample was diluted 10 times to a final binding buffer concentration of 50 mM Hepes pH 7.5, 140 mM NaCl, 1 mM EDTA, 1% Triton X-100, 0.1% Na-deoxycholate, 0.1% SDS, and 1 × PIC and stored at − 80 °C until use. Before use, the chromatin was precleared with 50 μl of Protein A or Protein G beads (Millipore, 16–157, Invitrogen, 10003D) for 1 h at 4 °C, followed by o/n incubation with 3 µg of antibodies against H3K9ac, H3K27ac, H3K4me1, RNAPII (RPB1) (respectively, ab4441, Abcam; ab4729 Abcam; ab8895 Abcam; RPB1-7G5 in-house). Next, protein A or protein G beads were added for a 3 h incubation at 4 °C. Subsequently, beads were washed 2 times with each of the buffers at 4 °C: binding buffer, buffer A (50 mM Hepes pH 7.5, 500 mM NaCl, 1 mM EDTA, 1% Triton X-100, 0.1% Na-deoxycholate, 0.1% SDS, 1 × PIC), buffer B (20 mM Tris pH 8, 1 mM EDTA, 250 mM LiCl, 0.5% NP40, 0.5% Na-deoxycholate, 1 × PIC), and TE buffer. DNA–protein complexes were eluted from beads, decrosslinked, and treated with proteinase K for 2 h at 43 °C. DNA fragments were purified using phenol–chloroform, precipitated, and analyzed by qPCR or used for sequencing.

ChIP samples were purified using Agencourt AMPure XP beads (Beckman Coulter) and quantified with Qubit (Invitrogen). ChIP-seq libraries were prepared from 0.5 to 10 ng of double-stranded purified DNA using the MicroPlex Library Preparation kit v2 (C05010014, Diagenode s.a., Seraing, Belgium), according to manufacturer’s instructions. In the first step, the DNA was repaired and yielded molecules with blunt ends. In the next step, stem-loop adaptors with blocked 5′ ends were ligated to the 5′ end of the genomic DNA, leaving a nick at the 3′ end. In the final step, the 3′ ends of the genomic DNA were extended to complete library synthesis and Illumina compatible indexes were added through a PCR amplification (7–10 cycles). Amplified libraries were purified and size-selected using Agencourt AMPure XP beads (Beckman Coulter) to remove unincorporated primers and other reagents. The libraries were sequenced on Hiseq 4000 as single end 50 base reads following Illumina’s instructions. Image analysis and base calling were performed using RTA 2.7.3 and bcl2fastq 2.17.1.14.

Reads were mapped to *Mus musculus* genome (assembly mm10) using Bowtie [34] v1.0.0 with default parameters except for -m 1 –best –strata”. RNAPII peaks were called using MACS2 v2.1.1. MACS2 was run with default parameters except for “-g mm -f BAM –broad –broad-cutoff 0.1”. H3K27ac, H4K4me1 and H3K9ac peaks were detected by SICER v1.1 [39] with the following parameters: Species: mm10. Threshold for redundancy allowed for ChIP reads: 1. Threshold for redundancy allowed for control reads: 1. Window size: 200 bps. Fragment size was set according to Homer v4.9.1 [40] MakeTagDirectory results. Effective genome size as a fraction of the reference genome of mm10: 0.77. Gap size: 600 bps. E.value for identification of candidate islands that exhibit clustering: 1000. False discovery rate controlling significance: 1 × 10^–2^. Peaks were annotated relative to genomic features using Homer v4.9.1 [40] (annotations got extracted from gtf file downloaded from Ensemble v90).

Heatmaps of our ChIP-seq data were generated using seqMINER v1.3.3 g [41]. The k-means clustering of H3K9ac data was performed using 10 clusters and a linear normalization. The same clusters were then used to visualize RNAPII and H3K27ac ChIPseq data. ChIPseq data visualization was performed using IGV v2.8.3 [42].

### ChIP-seq densities calculation

The number of aligned reads per region of interest was determined with seqMINER (Enrichment based method) using input BED files [41]. The end of each aligned read was extended to 200 bp by the program, in the direction of the read. Density values were defined as follows:

$$\text{Densities WT} = \text{median FC (SCA7/WT) intergenic}^* \times (\text{number of aligned reads in a region of interest/ length of the region of interest in Mb}) / \text{total number of aligned reads in the dataset} \times 10^{-8}$$

$$\text{Densities SCA7} = (\text{number of aligned reads in a region of interest/ length of the region of interest in Mb}) / \text{total number of aligned reads in the dataset} \times 10^{-8}$$

$$^*\text{median FC (SCA7/WT) intergenic: Normalization factor for WT dataset} = \text{median of [(number of aligned reads in intergenic region in SCA7/ length of the intergenic region in Mb) / total number of aligned reads in the dataset} \times 10^{-8})]/ [(\text{number of aligned reads in intergenic region in WT/ length of the intergenic region in Mb) / total number of aligned reads in the dataset} \times 10^{-8}]$$

For the analysis of H3K9ac and H3K27ac, the regions of interest corresponded to a window surrounding each transcription start site (TSS) from − 300 bp to + 300 bp (called TSS window). The H3K4me1 densities were calculated on the entire gene body [from the TSS to the transcription termination site (TTS)]. The RNAPII densities were calculated either on the TSS window or on the rest of the gene body (from TSS + 301 bp to TTS). Densities were calculated on genes which were defined as expressed and which had at least one annotated RNAPII peak.

### Establishing RNAPII pausing index (PI)

Pausing index was described as follows. Pausing index = 5′ read density / gene body density. We defined the 5′ regions of genes as the regions comprised between TSS-30 nucleotides (nt) and TSS + 300 nt. Gene body region was defined as the region comprised between TSS + 301nt and TTS-300nt. Pausing index was computed on Ensembl genes v90.

### Analysis of predicted superenhancers (SE) in the retina of adult WT mouse

To identify the superenhancers, the ROSE algorithm version 0.1 was applied with default parameters [43, 44] using the H3K27ac peaks identified by SICER. Peaks overlapping with ENCODE mm10 blacklist v1 [45] were filtered. TSS regions (Refseq TSS ± 2000 bp) were excluded. H3K27ac metagene representations of SEs were obtained as described [43] by applying the “bamToGFF” function of ROSE. We used the Galaxy platform with BEDTools (bedtools intersect intervals, − f = 0.5) [46] to determine the number of H3K9ac/H3K27ac peaks of each cluster that are more than 50% covered by the predicted superenhancers.

### Identification of putative enhancer RNAs (eRNAs)

We used a previously reported workflow [47] to identify reads from putative long non-coding RNAs synthesized from enhancers (eRNA). Briefly, we removed split-mapped reads and reads that overlapped (≥ 1 bp on the opposite strand) genes annotated by Ensembl (release 90) as: IG_C_gene, IG_C_pseudogene, IG_D_gene, IG_D_pseudogene, IG_J_gene, IG_LV_gene, IG_pseudogene, IG_V_gene, IG_V_pseudogene, Mt_rRNA, Mt_tRNA, polymorphic_pseudogene, processed_pseudogene, protein_coding, pseudogene, rRNA, transcribed_processed_pseudogene, transcribed_unitary_pseudogene, TR_C_gene, TR_D_gene, TR_J_gene, TR_J_pseudogene, TR_V_gene, unitary_pseudogene or unprocessed_pseudogene. We extended the region corresponding to those genes to 3 kb upstream of the transcription start site and 10 kb downstream of the transcript end site, in order to minimize signal from polymerase read-through from genic transcripts [48]. IntersectBed from BEDTools release 2.21.0 was used for this purpose. This overlap was performed on the opposite strand as the library preparation protocol we used to construct these RNA-seq libraries lead to sequence the strand generated during first strand cDNA synthesis. On those filtered RNA-seq reads, we then detected the location of putative eRNA as genomic regions enriched in RNA-seq reads. We thus used a method for ChIP-seq peak detection: we used MACS v1.4.2 [49] with the following parameters: –keep-dup = all –nomodel –nolambda -p 1e-4 –g mm. The parameter –shiftsize was set according to the value assessed by Homer v4.9.1 makeTagDirectory. Putative eRNA positions were compared to positions of H3K9ac and H3K27ac peaks. Putative eRNAs were annotated using Homer v4.9.1 annotatePeaks.pl with annotation extracted from Ensembl v90.

### Search for transcription factor (TF) binding motifs

Known TF binding motifs (JASPAR2018 CORE non-redundant motif database [50]) within genomic sequences of cluster 10 were searched using AME v.5.3.3 with default advanced options [51]. The genomic sequences of cluster 1–9 were used as negative control. The identified motifs were further filtered to obtain only the ones binding TF expressed in WT retina with an annotated RNAPII peak. Next, the overlap of these TFs with genes involved in photoreceptor cell differentiation (Amigo software) was performed and the transcription factors essential for photoreceptor cell differentiation and maintenance were identified. FIMO [52] motif scan was subsequently performed in order to confirm the number of occurrences of each motif.

### Gene ontology and network analysis

Gene ontology analysis was performed using DAVID v. 6.8 [53]. The STRING v.11.0 software was used to generate the protein interaction network (0.700 STRING confidence score).

### Photoreceptor specific genes dataset

We defined a subset of 95 photoreceptor specific genes using data mining (Additional file 1: Table S2). Photoreceptor specific genes were defined as retinal genes only expressed in photoreceptors based on transcriptomic and *in-situ* hybridization data from the publicly available protein atlas https://www.proteinatlas.org. 50 photoreceptor specific genes were retrieved from a public web page (http://retina.tigem.it) [54], 11 from [55] and 34 from [56].

### Housekeeping genes dataset

In order to build a robust list of housekeeping genes we merged two publicly available datasets [57, 58] containing, respectively, 373 and 3804 human housekeeping genes. We then used ENSEMBL to identify the mouse orthologs. The final list was composed of 3485 housekeeping genes overlapping with our RNA-seq (Additional file 1: Table S3).

### CRX direct transcriptional targets

The direct transcriptional target genes of CRX were defined as a gene set with a CRX binding site(s) and which expression is significantly altered in *Crx* knockout mice (Additional file 1: Table S4). To build such a dataset we first realigned and annotated the CRX ChIP-seq data from [59] in the genome assembly mm10. Realigned CRX peaks were annotated to the closest TSS of genes and led to the identification of 10,651 protein coding genes which have at least one CRX binding site. Next, differentially expressed genes in *Crx* knockout mice retina were obtained by merging the results of two independent studies [59, 60]. Microarray data from these studies were converted into the genome assembly mm10 using conversion tables available on http://www.affymetrix.com. To obtain a set of direct transcriptional targets of CRX, we then overlapped the genes harboring a CRX binding site(s) and the genes deregulated in the *Crx* knockout mice.

### NRL direct transcriptional targets

The direct transcriptional target genes of NRL were defined as a gene set with NRL binding site(s) and which expression is significantly altered in a *Nrl* knockout mice (Additional file 1: Table S5). To build such a dataset we first realigned and annotated the NRL ChIP-seq data from [61] in the genome assembly mm10. Realigned NRL peaks were annotated to the closest TSS of genes and led to the identification of 1603 genes. Next, differentially expressed genes in *Nrl* knockout mice retina were obtained by merging the result of three independent studies [60, 62, 63]. Microarray data from [60] were converted into the genome assembly mm10 as above. To obtain a set of direct transcriptional targets of NRL, we then overlapped the genes harboring NRL binding site(s) and the genes deregulated in the *Nrl* knockout mice.

### Developing retina datasets

Publicly available H3K27ac and H3K9ac ChIP-seq data from GSE87037 were reannotated to *Mus musculus* genome according to assembly mm10 using Bowtie v1.0.0 with default parameters [34]. Superenhancer coordinates, as defined in [64], were then converted into *Mus musculus* assembly mm10 (UCSC Table Browser), and used to cluster H3K27ac ChIP-seq (SeqMiner v1.3.4; clustering normalization Kmean linear; 15 clusters). These superenhancers were then divided into different groups according to the signal intensity between P0 and P21 H3K27ac ChIP-seq on a window covering the center of the superenhancer ± 5 kb.

### Experimental design and statistical analysis

All measurements were performed using distinct samples from age-matched WT and SCA7 mice. Data were analyzed using GraphPad Prism 8 and R. For a comparison of two groups, data were tested for normality using Shapiro’s test, and statistical analyses included two-tailed unpaired Student’s *t* test or Mann–Whitney U test. Other analyses were performed using ordinary one-way ANOVA, followed by *post-hoc* Tukey testing of pairwise comparisons, and hypergeometric test. Significance was established at p < 0.05. Further information is indicated in figure legends. Datasets used and/or analyzed during the current study are available from the corresponding author on reasonable request.

## Results

### Highly expressed photoreceptor identity genes are preferentially downregulated in SCA7 retina

To carry out an integrated analysis of transcriptomic and epigenomic changes in SCA7 retina, we used a new SCA7 knock-in mouse model which expresses mATXN7 harboring 140 glutamines (140Q) under *Atxn7* physiological promoter and recapitulates the major physiopathology traits of SCA7 [11]. The retinopathy of SCA7^140Q/5Q^ heterozygous mice was previously reported [11] to begin between 5 and 10 weeks of age, and to worsen until an almost complete loss of electroretinogram (ERG) activities at 20–24 weeks, correlating with strong downregulation of cone opsins and rhodopsin photopigment genes, important shortening of photoreceptor OS, and no other overt histological alteration or cell loss (Additional file 2: Fig. S1A). We aimed to take advantage of SCA7^140Q/140Q^ homozygous mice—in which mATXN7 replaces its wild type form in all SAGA complexes—to analyze unbiased relationships between gene deregulation and alteration of the HAT activity of SAGA complexes containing only the mutated form of ATXN7. The retinopathy of SCA7^140Q/140Q^ mice develops more rapidly, and the downregulation level of photopigment genes of 12.5-weeks SCA7^140Q/140Q^ corresponds to that of 22-weeks SCA7^140Q/5Q^ mice (Additional file 2: Fig. S1B). At 12.5-weeks, ERG activities and length of photoreceptor OS of SCA7^140Q/140Q^ mice were also highly reduced (Additional file 2: Fig. S1C-D).

**Fig. 1:**
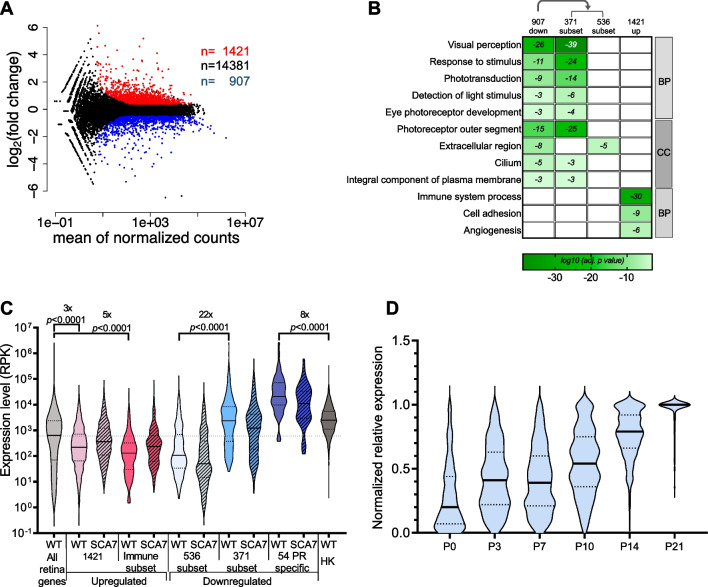
*Preferential* downregulation of highly expressed photoreceptor identity genes in SCA7. **A** MA-plot of RNA-seq analysis depicting differentially expressed protein coding genes in 12-week-old SCA7^140Q/140Q^ retina compared to age-matched WT. Red and blue dots represent upregulated (FC > 1.3; adj. p < 0.05) and downregulated (FC < 0.7; adj. p < 0.05) genes, respectively, and black dots correspond to non-deregulated genes. **B** Enrichment of Gene Ontology (GO) terms for biological processes (BP) and cellular components (CC) (log10(adjusted *p* value) ≤ 10^–3^) among the 907 downregulated genes and 1421 upregulated genes in SCA7 retina. A subset of 371 downregulated genes (out of 907) accounted for almost all retrieved vision GO terms, while the remaining 536 downregulated genes retrieved only one enriched GO term. **C** Violin plots comparing the expression levels of different subsets of and upregulated and downregulated genes in WT and SCA7 retina. In WT retina, the 371-subset of downregulated genes has higher expression level than the 536-subset. Among the 371-subset, 54 photoreceptor (PR) specific genes are highly expressed, as compared to housekeeping (HK) genes. In contrast, 187 upregulated genes associated to immune system process have very low expression level in WT retina, when compared to entire population of expressed retinal genes. Data were analyzed using Mann–Whitney test. **D** Violin plots showing the relative expression level of the subset of 371 genes downregulated in SCA7 and associated with vision functions, at different stages of retinogenesis in WT retina (RNA-seq data source from [64]). Expression level of each gene is normalized relative to its lowest (0) and highest (1) value across mouse retina development. P, post-natal day

We first performed total RNA-seq of WT and SCA7^140Q/140Q^ retina of 12.5-week-old animals to identify altered genes with functions of high significance for the retinopathy. From the 16,709 protein coding genes detected in WT retina, 907 had a significantly decreased expression in SCA7 retina, with fold change (FC) (SCA7/WT) < 0.7 (adjusted *p* value < 0.05) (Fig. 1A and Additional file 1: Table S6). The large majority (83%; 750/907) of these genes are known to be expressed in rod and cone photoreceptors (Additional file 2: Fig. S2A). Gene ontology (GO) enrichment analysis revealed the highest enrichment for biological processes (GO_BP) related to visual perception, phototransduction and eye photoreceptor development, as well as enrichment for cellular component (GO_CC) associated with OS, plasma membrane and cilium (Fig. 1B). Strikingly, all these enriched GO terms were determined by a subset of 371 downregulated genes, while the remaining 536 downregulated genes showed some low enrichment for GO_CC term related to extracellular region and are thus likely involved in diverse functions (Fig. 1B and Additional file 1: Table S6). The 371 genes have expression level 22 times higher than that of the remaining 536 downregulated genes in the WT adult retina (respectively, median RPK of 2269 versus 106; *p* < 0.0001), and notably include a subgroup of 54 photoreceptor specific genes expressed at an extremely high level compared to reference housekeeping genes (8 times higher at median RPK comparison: respectively, 21,026 versus 2493, *p* < 0.0001) (Fig. 1C). Interestingly, these 371 genes were found to be strongly upregulated during the late phases of photoreceptor differentiation and maturation (post-natal day 10–21) in WT retina (Fig. 1D) [64], highlighting their importance in the acquisition of photoreceptor identity. Finally, it is also noteworthy that as many as 71 of these downregulated genes code for proteins located in cilia and OS, and compose a large interconnected protein network of 168 interaction edges (*p* < 1.0 × 10^–16^) (STRING v.11.0) (Additional file 2: Fig. S2B and Additional file 1: Table S7). Western blot analysis confirmed that the expression of mature photoreceptor identity genes was also decreased at the protein level and occurred as early as 5 weeks of age in SCA7^140Q/140Q^ retina (Additional file 2: Fig. S2C). This 371-gene list includes the 22 photoreceptor genes previously shown to be downregulated in the retina of SCA7 transgenic mice [7, 8, 10, 11]. Our results thus largely extend the spectrum of gene downregulation and provide a molecular basis for the progressive shortening of OS and their lack of renewal in SCA7 mouse retina.

RNA-seq analysis also identified 1421 significantly upregulated genes with FC (SCA7/WT) > 1.3 (adjusted *p* value < 0.05) in SCA7^140Q/140Q^ retina that were highly enriched in GO_BP terms related to immune system process, and to a less extent to cell adhesion and angiogenesis (Fig. 1A, B and Additional file 1: Table S8). Interestingly, 187 genes related to immune system process compose a large interconnected protein network of 1534 interaction edges (*p* value: 4.60 × 10^–135^) (Additional file 2: Fig. S3A). These 187 genes, which include activated microglial and glial genes, have very low expression level in the WT adult retina when compared to the entire population of expressed retinal genes (5 times lower at median RPK comparison: respectively, 130 RPK versus 631, *p* < 0.0001) (Fig. 1C). Consistent with microglia and glia activation, IBA1 and GFAP proteins, respectively, are readily detected in SCA7 retina, compared to their low basal level in WT (Additional file 2: Fig. S3B). The activation of glial response also occurs in SCA7^140Q/5Q^ heterozygote retina between 19 and 27 weeks of age (Additional file 2: Fig. S3C), which is noticeably observed about 10 weeks after the onset of the retinopathy.

In summary, the most striking changes in gene expression in SCA7^140Q/140Q^ retina are the downregulation of highly expressed genes involved in mature photoreceptor identity, which explains the early deficit in photoreceptor function and OS maintenance. The activation of microglial and glial genes associated to immune response appears as a late event in the disease process. Since downregulation of photoreceptor identity genes has early and strong impact on photoreceptor function and morphology in SCA7, subsequent experiments were primarily aimed at identifying transcriptional and epigenomic alterations causing a change in expression of these genes.

### SCA7 is not causing a global CRX and NRL dysfunction per se

Given that CRX and NRL are main regulators of photoreceptor genes, we explored how CRX and NRL dysfunction could contribute to gene downregulation. In SCA7^140Q/140Q^ retina, the level of *Crx* and *Nrl* mRNAs was reduced by 33% (*p* = 2.4 × 10^–12^) and 66% (*p* = 3.3 × 10^–61^), respectively, compared to WT (Additional file 1: Table S6). However, since mutant heterozygous mice expressing one allele of *Crx* or *Nrl* showed no retinal phenotype, the decreased level of these factors in SCA7 retina appears unlikely to account for downregulation of their target genes. Furthermore, timeline analysis in different SCA7 mouse models showed that photopigment gene mRNAs were already decreased at times when *Crx* or *Nrl* mRNA expression levels were not yet affected, suggesting that the primary cause of downregulation is not due to the decreased amount of these transcription factors [8, 10, 65] (Additional file 2: Fig. S4A). Using an in silico approach, we established a list of CRX and NRL direct transcriptional targets using published datasets; direct target gene was defined as a gene associated with CRX or NRL binding site(s) as established by ChIP-seq experiments, and showing altered expression in *Crx* or *Nrl* knockout mice (Methods and Additional file 1: Table S4-S5). We then compared the list of target genes to the rod photoreceptor genes downregulated in SCA7^140Q/140Q^ retina. Among a total of 1486 target genes (805 CRX targets, 376 NRL targets and 305 CRX-NRL common targets), 249 targets (< 17%) were downregulated in SCA7^140Q/140Q^ (Additional file 2: Fig. S4B), including 197 genes involved in photoreceptor cell identity. Given that a minority of CRX and NRL target genes are downregulated in SCA7 retina, the results suggest that mATXN7 does not cause global CRX and NRL dysfunction per se, and that alteration of additional regulatory mechanisms likely determines the propensity of a subset of CRX and NRL target genes to be downregulated.

### H3K9 and H3K27 acetylation of gene promoters are widely impaired in SCA7 retina

**Fig. 2:**
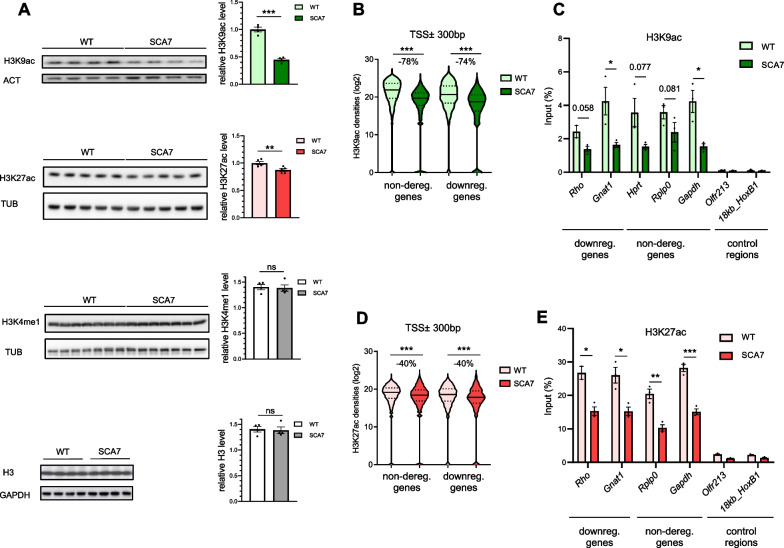
Genome-wide analysis H3K9 and H3K27 acetylation at gene promoters in SCA7 retina. **A** Western blot analyses showing the significant decrease of H3K9ac and H3K27ac levels in SCA7 retina compared to WT, while H3K4me1 and unmodified H3 levels are not affected. Data are normalized to the level of control proteins (actin (ACT), tubulin (TUB) and GAPDH), expressed as mean ± SEM (*n* = 4–7 mice/genotype) and analyzed using two-tailed Student’s *t*-test. **B** Violin plots showing the reduction of H3K9ac densities on regions ± 300 bp around the transcription start site (TSS ± 300 bp) of non-deregulated and downregulated genes in SCA7 retina, compared to WT. **C** ChIP-qPCR analysis showing the decrease of H3K9ac occupancy (% of the input) on the TSS region of representative downregulated and non-deregulated genes in SCA7 retina compared to WT. Silent regions are used as controls. **D** Violin plots showing the reduction of H3K27ac densities on the TSS ± 300 bp regions of non-deregulated and downregulated genes in SCA7 retina, compared to WT. **E** ChIP-qPCR analysis showing the decrease of H3K27ac occupancy on the TSS region of representative downregulated and non-deregulated genes in SCA7 retina compared to WT. Silent regions are used as controls. Data in **B** and **D** were analyzed using Mann–Whitney test. Data in C and E are normalized as percentage of input DNA signal, expressed as mean ± SEM (*n* = 3 mice/genotype) and analyzed using two-tailed Student’s *t*-test. *p < 0.05; **p < 0.01; ***p < 0.001; ns non-significant

Previous analyses of a limited number of gene promoters in SCA7 transgenic mice have led to conflicting results regarding their acetylation defect [18, 19]. Therefore, we performed a genome-wide analysis of gene promoter acetylation to determine how epigenetic alterations are associated with gene downregulation in the new SCA7 knock-in mouse model. First of all, we compared the bulk level of different post-translationally modified H3 in SCA7 and WT retina by Western blot. The analysis showed that the level of H3K9ac and H3K27ac were, respectively, highly and modestly decreased in SCA7 retina [55% reduction for H3K9ac (*p* < 0.001) and 12.5% for H3K27ac (*p* < 0.01)], while H3K4 monomethylation (H3K4me1) and unmodified H3 were not affected (Fig. 2A). As rod photoreceptors largely constitute the major retinal cell type [66], their epigenetic profiles in the retina are dominant over other cell types. We then performed ChIP-seq analysis to determine the densities of H3K9ac and H3K27ac at gene promoter regions (transcription start site (TSS) ± 300 bp) (Methods and Additional file 2: Fig. S5). H3K9ac densities at gene promoter regions were much lower in SCA7 retina compared to WT and similarly decreased for downregulated and non-deregulated genes (fold reduction of -74% and -78%, respectively; *p* < 0.001) (Fig. 2B). Using additional 12.5-week-old animals, ChIP-qPCR analysis confirmed the lower density of H3K9ac at promoter regions of representative downregulated photoreceptor genes and non-deregulated housekeeping genes in SCA7 retina (Fig. 2C). Similarly, the densities of H3K27ac at gene promoter regions were reduced in SCA7 retina for downregulated and non-deregulated genes, although to a lesser extent than for H3K9ac (fold reduction of -40% for H3K27ac for both gene categories, *p* < 0.001), and H3K27ac reduced densities were confirmed for representative genes using ChIP-qPCR analysis (Fig. 2D, E). In contrast, the densities of H3K4me1 along the gene bodies of expressed genes were slightly increased in SCA7 retina (fold increase of + 8.7% for downregulated and + 9.4% for non-deregulated; *p* < 0.001) (Additional file 2: Fig. S6). Taken together, the results indicate that H3K9ac and H3K27ac densities at most gene promoter regions show, respectively, a strong and a mild reduction in SCA7 retina, consistent with the decreases of bulk H3K9ac and H3K27ac quantified on Western blot. However, these reductions do not necessarily cause change in gene expression.

### Photoreceptor identity genes downregulated in SCA7 have low RNAPII pausing

**Fig. 3:**
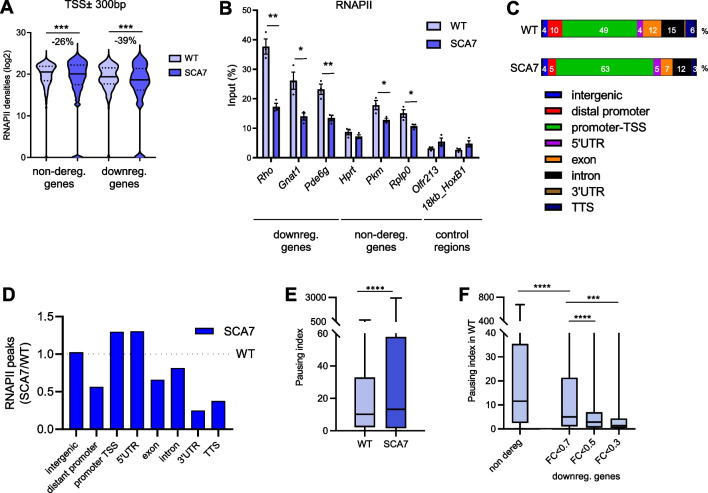
SCA7 downregulated genes have low RNAPII pausing. **A** Violin plots showing the decrease of RNAPII densities on TSS ± 300 bp regions of non-deregulated and downregulated genes in SCA7 retina, compared to WT. **B** ChIP-qPCR analysis showing the decrease of RNAPII occupancies (% of the input) on the TSS region of representative downregulated and non-deregulated genes in SCA7 retina compared to WT. Silent regions are used as controls. **C** Comparison of genomic distribution of RNAPII peaks in WT and SCA7 retina. Data are displayed as percentage of peaks annotated to each region relative to total number of peaks. Distant promoter (− 20 kb to − 1 kb relative to the TSS), promoter-TSS (− 1 kb to + 100 bp at the TSS), and TTS (− 100 bp to + 1 kb relative to the TTS). **D** Graph representing the ratio of RNAPII peak distribution in different genic and intergenic regions in SCA7 and WT retina. **E** Graph showing the comparison of RNAPII pausing index in WT and SCA7 retina for all expressed protein coding genes. **F** Graph showing the comparison of RNAPII pausing index at basal level in WT retina for non-deregulated gene sets and SCA7 downregulated genes with different fold change (FC). Data in **A**, **E** and **F** were analyzed using Mann–Whitney test. Data in **B** are normalized as percentage of input DNA signal, expressed as mean ± SEM (*n* = 3 mice/genotype) and analyzed using two-tailed Student’s *t*-test. *p < 0.05; **p < 0.01; ***p < 0.001, ****p < 0.0001

Earlier studies showed that disruption of SAGA HAT activity in yeast results in an overall decrease in H3K9ac at gene promoters and leads to a reduction in RNAPII-driven transcription [67, 68]. Therefore, we analyzed the occupancy of RNAPII at gene promoter regions by ChIP-seq in SCA7 retina. Consistent with the global reduction of H3K9ac, RNAPII occupancy was significantly lower compared to WT, for downregulated and non-deregulated genes (fold reduction of − 39% and − 26%, respectively; *p* < 0.001), and these reductions were confirmed by ChIP-qPCR analysis on additional animals (Fig. 3A, B). The data suggest that histone H3 hypoacetylation and decreased RNAPII occupancy at gene promoters are insufficient to alter the steady-state expression level of most genes.

We then analyzed the relative distribution of annotated RNAPII peaks in different genic (distal promoter, promoter-TSS, 5′UTR, exons, introns, 3’UTR and TTS) and intergenic regions. This provided a proxy for RNAPII dynamics along expressed genes. As expected, RNAPII peaks had the highest occupancy at promoter-TSS (− 1 kb to + 100 bp flanking the TSS) compared to other gene regions, in both WT and SCA7 retina (Fig. 3C). However, the distribution of RNAPII peaks at promoter-TSS and at 5’UTR was higher in SCA7 retina compared to WT (Fig. 3D). Conversely, the distribution of RNAPII peaks on downstream genic regions (intron, exon, 3′UTR and TTS) was lower in SCA7. These results suggest that RNAPII tends to stall at TSS region of most expressed genes in SCA7 retina. Alterations of RNAPII distribution within gene loci prompted us to analyze the RNAPII pausing of all expressed genes. Pausing of RNAPII at TSS shortly after transcription initiation and its release for transcription elongation constitutes a regulatory step for a majority of expressed genes [69]. As a consequence of pausing, the RNAPII occupancy at TSS is usually much higher than on the gene body. We thus estimated the RNAPII pausing by calculating the pausing index (PI), which is defined as the ratio between densities of RNAPII at the TSS versus body of the same gene [70]. RNAPII PI was higher in SCA7 retina than in WT for all expressed genes (1.3 fold increase of median; 13.2 versus 10.2, *p* < 0.0001) (Fig. 3E), further suggesting that the RNAPII dynamics is globally affected in SCA7 retina. However, it was noteworthy that at basal level in WT retina, the RNAPII PI of genes downregulated in SCA7 was significantly lower than the RNAPII PI of the non-deregulated genes (2.3 fold decrease of median; 11.53 versus 4.99 *p* < 0.0001) (Fig. 3F). Furthermore, the most downregulated genes (with SCA7/WT FC of < 0.5 and < 0.3), which include highly expressed photoreceptor identity genes, had even lower RNAPII PIs at basal level. For instance, genes with SCA7/WT FC of < 0.3 had RNAPII PI 8.3 fold lower than the RNAPII PI of the non-deregulated genes (median 11.53 versus 1.39, *p* < 0.0001). This suggests that photoreceptor identity genes predisposed to downregulation in SCA7 are characterized by distinct RNAPII pause release and transcriptional dynamics under WT conditions.

### An unusually broad H3K9ac profile marks photoreceptor identity genes and is impaired in SCA7

**Fig. 4:**
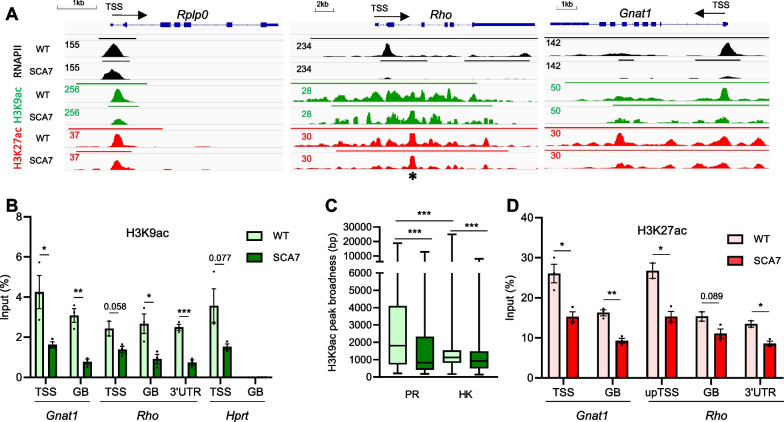
Atypically broad H3K9 acetylation is deposited at photoreceptor identity gene loci and is altered in SCA7 retina. **A** Genome browser tracks depicting RNAPII, H3K9ac and H3K27ac distributions at a representative housekeeping gene (*Rplp0*) and two photoreceptor specific genes (*Rho* and *Gnat1*) in WT and SCA7 retinas. In WT retina, RNAPII, H3K9ac and H3K27ac show characteristic narrow peaks in the TSS region of *Rplp0*. In contrast, broad signals of H3K9ac, H3K27ac and RNAPII are found throughout the entire gene bodies of *Rho* and *Gnat1.* In SCA7 retina, peak height and peak broadness as illustrated by the called peaks (top straight bar) of RNAPII, H3K9ac and H3K27ac are decreased at *Rho* and *Gnat1* genes. * indicate saturated peak in *Rho* gene due to repetitive sequences. **B** ChIP-qPCR analysis shows H3K9ac occupancy on the TSS, gene body (GB) and 3′UTR regions of *Rho* and *Gnat1* genes, as well as occupancy on the TSS, but not on the GB of the housekeeping *Hprt* gene in WT retina. In SCA7 retina, H3K9ac occupancy is globally decreased on *Rho* and *Gnat1* gene loci and on the TSS of *Hprt* gene. Data are normalized as a percentage of input DNA, expressed as mean ± SEM (*n* = 3 mice/genotype) and analyzed using two-tailed Student’s *t*-test. **C** Box plots depicting the broadness (in base pairs (bp)) of H3K9ac peaks on photoreceptor specific genes (n = 95) and on housekeeping genes (n = 3485) in WT and SCA7 retina. Data were analyzed using Mann–Whitney test. **D** ChIP-qPCR analysis shows H3K27ac occupancy along the *Rho* and *Gnat1* gene loci in WT retina and its decrease in SCA7 retina; (upTSS, upstream of TSS). Data are normalized as a percentage of input DNA, expressed as a mean ± SEM (*n* = 3 mice/genotype) and analyzed using two-tailed Student’s *t*-test. *p < 0.05; **p < 0.01; ***p < 0.001

To decipher why highly expressed photoreceptor identity genes with low RNAPII pause are prone to downregulation in SCA7 retina, we examined the distribution of H3K9ac along gene loci in the WT retina. As expected [5], H3K9ac presented canonical narrow profiles of densities on the TSS of most genes, as shown for the housekeeping *Rplp0* (Fig. 4A) and *Hprt* genes (Additional file 2: Fig. S7A). Interestingly, photoreceptor identity genes vulnerable to downregulation in SCA7 retina such as *Rho* and *Gnat1*, displayed unusually broad H3K9ac profiles spreading from upstream regulatory regions throughout gene bodies. ChIP-qPCR analysis confirmed that *Rho* and *Gnat1* gene loci had broad H3K9ac occupancies (on TSS, gene body and 3′UTR) in WT mice, while H3K9ac densities were present on the TSS, but not on the gene body of housekeeping genes (Fig. 4B). Compared to WT, the ChIP-seq data showed a reduction of H3K9ac profiles along the *Rho* and *Gnat1* loci in SCA7 retina (Fig. 4A), and ChIP-qPCR analysis of additional SCA7 animals further confirmed lower H3K9ac densities along the *Rho* and *Gnat1* loci (Fig. 4B). To determine the cell-type specificity of the broad H3K9ac profiles, we compared the broadness of H3K9ac peaks annotated to genomic loci of photoreceptor specific genes and housekeeping genes (Additional file 1: Table S2-S3). In WT retina, the broadness of H3K9ac peaks of photoreceptor specific genes was 2.3 fold higher (p < 0.001) than for housekeeping genes (*p* < 0.001) (Fig. 4C). Furthermore, in SCA7 retina, the broadness of H3K9ac peaks was strongly reduced (− 38%; *p* < 0.001) for photoreceptor specific genes, and much less reduced (− 13%; *p* < 0.001) for housekeeping genes. Interestingly, the broad H3K9ac profiles at *Rho* and *Gnat1* loci correlated with a similar broad distribution of H3K27ac (Fig. 4A). As for H3K9ac, the densities of H3K27ac signal along the *Rho* and *Gnat1* loci were reduced in SCA7 retina (Fig. 4A) and this reduction was confirmed by ChIP-qPCR analysis on additional animals (Fig. 4D). Markedly, the alteration of the broad H3K9ac and H3K27ac profiles along *Rho* and *Gnat1* gene loci was associated with a strong reduction of RNAPII occupancy (Fig. 4A). Taken together, the results indicate that photoreceptor identity gene loci harbor unusually broad H3K9 acetylation profiles that coincide with broad H3K27 acetylation and that both acetylation profiles are altered in the SCA7 retina.

Of note, the H3K9ac, H3K27ac and RNAPII profiles of upregulated genes expressed in microglial and glial cells (e.g. *Trem2*, *Aif1* and *Gfap*) were barely detected, indicating that epigenetic profiles could not be covered for genes expressed in low-abundance retinal cell types (Additional file 2: Fig. S7B). More than half of the 187 immune genes had no detectable H3K9ac peaks, hampering further correlative analysis of epigenetic profiles versus gene upregulation for this class of genes.

**Fig. 5:**
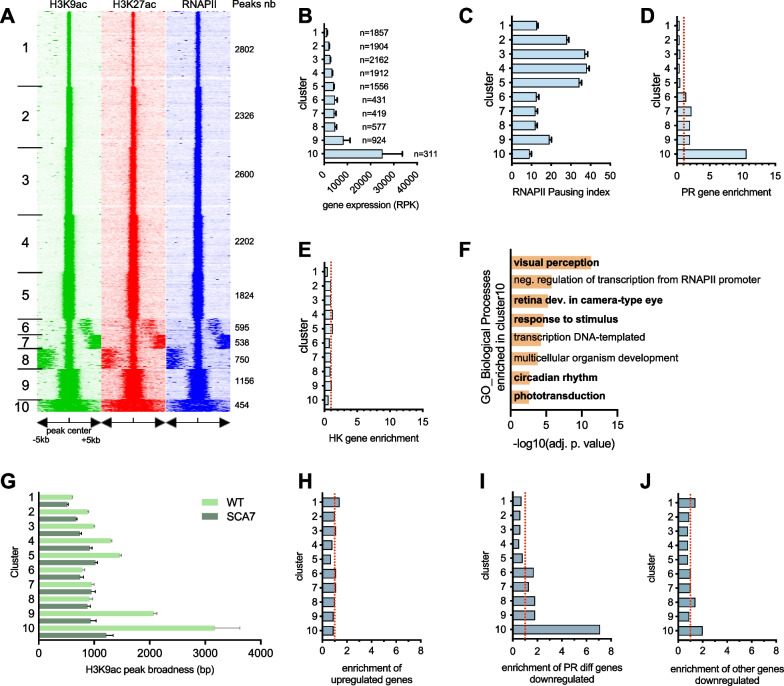
Broad H3K9ac defines a category of photoreceptor genes vulnerable to downregulation in SCA7. **A** Heatmap integrating the genome-wide profiles of H3K9ac, H3K27ac and RNAPII densities in WT retina. The window corresponds to the center of H3K9ac peaks ± 5 kb. Heatmaps were obtained from seqMINER with 10 clusters corresponding to different epigenomic states. **B, C** Graph representing the expression level (**B**) and RNAPII pausing index (**C**) of protein coding genes annotated to each cluster in WT retina. n, number of protein coding genes per cluster. Data presented as mean ± SEM and are analyzed using one-way ANOVA followed by Tukey *post-hoc* test (expression level: F_(9, 12072)_ = 16.37, *p* < 0.0001; pausing index, F_(9, 14075)_ = 99.63, *p* < 0.0001). **D, E** Graphs representing the enrichment of photoreceptor specific (PR) (**D**) and housekeeping (HK) (**E**) genes annotated to each cluster. The analysis concerns the distribution of 95 PR genes and 3485 HK genes across all clusters, and bars refer to the ratio of observed versus expected number of genes in each cluster normalized to 1 (dashed red line). The enrichments are 11 × more PR specific genes (*p*_cluster10_ = 5.1 × 10^–23^, hypergeometric test) and 1.6 × less HK genes (*p*_cluster10_ = 4.9 × 10^–6^) than expected in cluster 10. **F** Gene ontology (GO) analysis of cluster 10. Visual perception and other photoreceptor related processes (bold) are the most significantly enriched biological processes (adj. *p* value < 0.01) associated with protein coding genes in cluster 10. **G** Graph comparing the broadness [in base pairs (bp)] of H3K9ac peaks in each cluster in WT and SCA7 retinas. Comparison WT versus SCA7 of cluster 10 using Mann–Whitney test, *p* < 0.0001. **H–J** Graph representing the enrichment per cluster of SCA7 upregulated genes (**H**), 371 SCA7 downregulated photoreceptor identity genes (**I**) and 536 other downregulated genes (**J**). The analysis concerns the distribution of each gene category across all clusters, and bars refer to the ratio of observed versus expected number in each cluster normalized to 1 (dashed red line). The enrichments are 7.1 × more photoreceptor identity genes (**I**) than expected in cluster 10 (*p*_*cluster10*_ = 5.6 × 10^–33^, hypergeometric test)

To delineate the relationship between the broad H3K9ac profile, H3K27ac mark, RNAPII occupancy and gene expression, we used seqMINER [41] to classify the broadness of H3K9ac at the genome-wide level and in an unbiased manner. Briefly, the density of H3K9ac was calculated in a 10 kb window with the peak reference coordinate at the center, and clustered using a k-means clustering (Fig. 5A). Ten distinct clusters were obtained. Clusters 1 to 5, which gather the large majority (77%) of H3K9ac peaks, showed canonical sharp H3K9ac profiles related to most protein expressed genes of retina (Fig. 5B). In contrast, clusters 6 to 10 revealed broader and more complex depositions. In particular, H3K9ac deposition in cluster 10 (representing only 3% of H3K9ac peaks) was observed on the entire 10 kb window. In addition, genomic regions characterized by broad deposition of H3K9ac were also associated with broad deposition of H3K27ac marks and extensive RNAPII occupancy (Fig. 5A), as already illustrated by the broad epigenomic profiles observed for *Rho* and *Gnat1* in Fig. 4A.

Interestingly, protein coding genes associated to cluster 10 were expressed in the WT retina at much higher levels than genes associated to other clusters (cluster 10 vs each one of other clusters, *p* < 0.0001) (Fig. 5B) and coincided with the lowest RNAPII pausing index (Fig. 5C). Moreover, they were enriched in photoreceptor specific identity genes with 11 times more genes than expected (hypergeometric distribution: *p*_cluster10_ = 5.1 × 10^–23^) (Fig. 5D), whereas housekeeping genes showed impoverishment among genes associated to cluster 10, compared to expected distribution (1.6 times lower, *p*_cluster10_ = 4.9 × 10^–6^) (Fig. 5E). Consistently, only genes of cluster 10 showed high enrichment for GO terms related to vision pathways (Fig. 5F). This cluster included 18 photoreceptor identity genes that are among the top 100 most rod expressed genes in WT retina [63] and that were downregulated in SCA7: *Cnga1*, *Cngb1*, *Crx*, *Gnat1*, *Gnb1*, *Grk1*, *Guca1a*, *Guca1b*, *Nrl*, *Pde6b*, *Prph2*, *Rcvn*, *Reep6*, *Rho*, *Rom1*, *Sag1*, *Slc24a1*, *Unc119*. This unbiased, genome-wide analysis provides further evidence that broad H3K9ac and H3K27ac profiles define a class of photoreceptor identity genes that are highly expressed and exhibit a low RNAPII pause.

To further determine how the broad H3K9ac and H3K27ac profile correlates with gene expression levels and how it is established at the loci of photoreceptor identity genes, we analyzed published epigenetic datasets for H3K9ac and H3K27ac established during retinal development [64]. We selected 8 photoreceptor identity genes with a wide range of expression level in WT retina [63] and downregulation in SCA7. The 8 genes show a concomitant broad deposition of H3K9ac and H3K27ac that progressively increased during rod differentiation and paralleled the increase of mRNA levels (Additional file 2: Fig. S8). This indicates that temporal increase in H3K9 and H3K27 acetylation correlates with higher expression of photoreceptor identity genes.

Next, we compared the broadness of annotated H3K9ac peaks at each cluster between WT and SCA7. The broadness of H3K9ac peaks in cluster 10 was strongly reduced in SCA7 retina [by 62% compared to WT (Mann–Whitney test, *p* < 0.0001)] (Fig. 5G), suggesting that genes associated with cluster 10 may be more vulnerable to deregulation in SCA7. To test this possibility, we analyzed the cluster distribution of the up and downregulated genes in SCA7. The upregulated genes were evenly distributed among the ten clusters and show no enrichment (Fig. 5H). In contrast, downregulated genes were enriched in cluster 10. Moreover, downregulated photoreceptor identity genes were highly enriched in cluster 10 compared to other clusters, with 7.1 times more genes than expected (hypergeometric distribution: *p*_cluster10_ = 5.6 × 10^–33^) (Fig. 5I), and compared to the other downregulated genes (Fig. 5J). Therefore, the results suggest that photoreceptor identity genes harboring a broad H3K9ac profile are more vulnerable to H3K9ac alteration and downregulation in the SCA7 retina.

### Broad H3K9 acetylated genomic regions harbor binding sites for photoreceptor specific transcription factors and superenhancer features

**Fig. 6:**
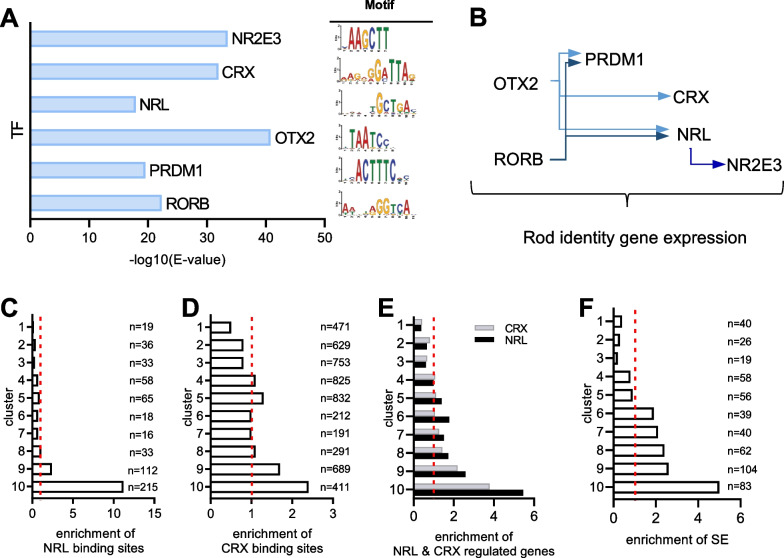
Cluster 10 harbors motifs for photoreceptor-specific transcription factors and cell-type specific enhancer features. **A** Graph showing the enrichment of AME predicted DNA motifs in genomic sequences of cluster 10, compared to sequences in clusters 1–9. These motifs (right panel) are consensual for transcription factors involved in photoreceptor cell fate. Left panel shows the corresponding DNA motif sequences. **B** Regulatory network composed of transcription factors involved in the development and differentiation of rod photoreceptors. **C, D** Graphs representing the enrichment of NRL (C) and CRX (D) binding sites in the genomic sequences of each cluster. The analysis concerns the distribution of 605 NRL and 5304 CRX binding sites across all clusters, and bars refer to the ratio of observed versus expected number of binding sites in each cluster normalized to 1 (dashed red line). The enrichment is 11 × more NRL binding sites (*p*_cluster10_ = 9.2 × 10^–180^, hypergeometric test) and 2.4 × more CRX binding sites (*p*_*cluster10*_ = 1.9 × 10^–115^) in cluster 10 than expected. **E** Graph representing the enrichment of NRL- and CRX-regulated genes in each cluster. The analysis concerns the distribution of 718 NRL- and 1048 CRX-regulated genes across all clusters, and bars refer to the ratio of observed versus the expected number of genes in each cluster normalized to 1 (dashed red line). **F** Graph representing the enrichment of ROSE predicted super-enhancer (SE) covering by more than 50% the H3K9ac genomic sequences of each cluster. The analysis concerns the distribution of 268 predicted SE across all clusters, and bars refer to the observed versus expected number of SE in each cluster normalized to 1 (dashed red line). The enrichment is 5 × more SE in cluster 10 than expected (*p*_*cluster10*_ = 4.2 × 10^–35^, hypergeometric test)

Given the enrichment of photoreceptor identity genes associated with the broad H3K9ac profiles, we asked whether the 10 kb genomic regions in cluster 10 are enriched in DNA binding sites for cell-type specific transcription factors. Using the motif enrichment software AME [51], 6 different DNA motifs consensual for transcription factors involved in photoreceptor cell differentiation and maintenance [15] were found to be enriched in cluster 10 when compared to genomic sequences of other clusters (Fig. 6A). These include CRX, NRL and NR2E3, which is a direct target of NRL and co-activator of rod photoreceptor identity genes [71, 72]. The three other factors, RORB, OTX2 and PRDM1, act at early steps in the acquisition of photoreceptor cell identity during development and compose a regulatory network with CRX, NRL and NR2E3 (Fig. 6B) [15, 16]. Using the FIMO algorithm [52], we estimated the occurrence of these 6 different motifs in cluster 10. The analysis showed that 92% (455 out of 495) of genomic sequences in cluster 10 contain at least one of these motifs, and have on average a co-occurrence of 6 motifs (Additional file 1: Table S9). The analysis indicates that broad H3K9ac profiles mark genomic sequences containing motifs for transcription factors involved in photoreceptor cell fate.

Given our previous results showing that a subset of CRX and NRL target genes are preferentially downregulated in SCA7 (Additional file 2: Fig. S4B), and that cluster 10 is enriched in downregulated photoreceptor genes, we determined to what extent broad H3K9ac profiles are related to CRX and NRL. To this end, we used public datasets of experimental NRL and CRX ChIP-seq [59, 61] and examined whether genomic sequences in cluster 10 are actually bound by these two transcription factors (Fig. 6C, D). Compared to clusters 1–9, cluster 10 showed the highest enrichment in NRL and CRX binding sites. In particular, NRL binding sites in cluster 10 were 11 times more frequent than expected (*p*_cluster10_ = 9.2 × 10^–180^) and represented 36% (215 out of 605) of all NRL binding sites of the genome. CRX binding sites, which were found more abundant in the genome by ChIP-seq, showed a 2.4 fold enrichment in cluster 10 (*p*_*cluster10*_ = 1.9 × 10^–115^ for CRX). Consistently, cluster 10 was highly enriched in NRL- and CRX-regulated genes (*p*_*cluster10*_ = 1.0 × 10^–45^ for NRL and *p*_*cluster10*_ = 1.1 × 10^–40^ for CRX) compared to other clusters (Fig. 6E). Together, the results point to a subset of CRX- and NRL-regulated genes associated with broad H3K9 and H3K27 acetylation that showed vulnerability to hypoacetylation and downregulation in the SCA7 retina.

Large genomic regions containing wide spreading of H3K27ac and binding sites for cell-type specific transcription factors typically define clusters of cell-type specific enhancers or superenhancers [43, 73]. However, the presence of H3K9ac mark at these superenhancers is less characterized. Therefore, we analyzed our H3K27ac ChIP-seq data with the ROSE algorithm [43] for prediction of superenhancers, and identified 268 such putative enhancers (mean size of 81 kb) in the retina of adult WT mice (Additional file 1: Table S10). Then, in each of the 10 clusters, we looked for H3K9ac/H3K27ac concomitant peaks that are more than 50% covered by the predicted superenhancers. The analysis revealed 83 H3K9ac/H3K27ac peaks in cluster 10 associated with predicted superenhancers (Fig. 6F), resulting in the highest enrichment in predicted superenhancers [5 times more than expected (*p*_*cluster10*_ = 4.2 × 10^–35^)], when compared to the other clusters. Among the downregulated photoreceptor identity genes associated to clusters 6–10, 32% of them were in the vicinity of a predicted SE (mediane distance of gene TSS to the SE center of 34 kb). This includes highly expressed photoreceptor identity genes such as *Rho*, *Gnat1*, *Gnb1*, *Grk1*, *Slc6a6*, *Rcvrn*, *Pde6a*, which are among the 100 most expressed genes of the mouse retina in our RNA-seq data.

### Enhancer-associated lncRNAs transcription at photoreceptor identity gene loci is impaired in SCA7 retina

Activation of enhancers and superenhancers is often associated with the transcription of a subset of lncRNAs, referred as eRNAs [26, 27]. We then asked whether loci marked by broad H3K9/H3K27 acetylation represent actively transcribed enhancer regions. Using a dedicated workflow, we found that 4301 putative lncRNAs were expressed in WT retina (Additional file 2: Fig. S9A). 282 putative lncRNAs were transcribed in genomic regions covered for more than 50% by broad peaks of H3K27ac and H3K9ac marks, suggesting that they are associated with enhancers and thus hereafter named putative eRNAs (Additional file 1: Table S11). Interestingly, 24 of these H3K27ac/H3K9ac-associated putative eRNAs were annotated to the highly expressed rod specific genes (examples shown in Additional file 2: Fig. S9B), and among these loci all except 4 were associated to cluster 10 (Table 1). We selected 8 putative eRNAs annotated to expressed rod specific genes (*Rho, Gnat1*, *Gnb1*, *Grk1*, *Guca1b*, *Plpp2*, *Sag* and *Unc119* gene) of cluster 10 for further validation using RT-qPCR with tagged strand specific primers [33]. The latter allowed strand specific analysis of lncRNAs transcribed antisense to the annotated genes, a frequent feature of mammalian lncRNAs [74]. RT-qPCR analysis showed that all selected putative eRNAs were readily co-expressed with their cognate mRNAs in purified mouse photoreceptors samples, but not in cerebellar samples used as controls (Additional file 2: Fig. S9C). Importantly, the expression levels of putative eRNAs and cognate *Rho*, *Gnat1*, *Gnb1*, *Grk1*, *Guca1b*, *Plpp2*, *Sag* and *Unc119* mRNAs increased correlatively and temporally during rod photoreceptor differentiation from P5, P10 and P21 in WT retina (Additional file 2: Fig. S10). Moreover, the increased expression of these putative eRNAs coincides with the increased broadness of H3K9ac and H3K27ac on the corresponding genomic regions as shown in Additional file 2: Fig. S8, further suggesting that these putative lncRNAs have features of eRNAs.

**Table 1:** Deregulated putative eRNAs annotated to photoreceptor specific genes of SCA7 mouse retina

Putative mouse eRNA			SCA7/WT expression in mouse retina			
Annotated PR specific gene	eRNA length [nt]	Cluster 10	Putative eRNA		mRNA	
			log2[FC]	adj. *p* value	log2[FC]	adj. *p* value
*Aipl1*	10,702	Yes	− 0,09	7,69E-01	− 0,16	6,14E-02
***Arl4d***	16,798	Yes	− 0,60	7,93E-04	− 0,78	5,27E-47
***Cnga1***	5168	Yes	− 0,58	2,54E-02	− 0,97	2,89E-38
***Cngb1***	7060	Yes	− 0,64	2,25E-03	− 0,62	1,22E-11
***Crb1***	5376	–	− 0,61	2,68E-02	− 0,26	2,00E-03
***Crb2***	7515	Yes	− 0,75	1,73E-03	− 0,32	6,54E-03
***Fam161a***	11,761	Yes	− 0,58	4,94E-03	− 0,59	3,67E-10
***Gnat1***	25,096	Yes	− 1,97	1,84E-88	− 1,75	2,79E-67
***Gnb1***	8377	Yes	− 1,14	1,04E-05	− 1,31	7,31E-48
*Gngt1*	7490	–	− 0,38	1,38E-01	− 1,06	3,50E-19
***Grk1***	12,836	Yes	− 1,54	5,72E-22	− 0,93	1,14E-34
***Guca1b***	9997	Yes	− 2,76	5,92E-36	− 1,64	1,49E-56
*Heg1*	10,544	Yes	− 1,02	4,92E-05	0,66	7,32E-09
***Pcbp4***	5495	–	− 1,12	1,98E-16	− 0,51	1,38E-06
***Pde6b***	12,407	Yes	− 0,71	5,52E-06	− 1,16	2,46E-46
***Plpp2***	7475	Yes	− 1,59	4,06E-06	− 1,13	3,60E-21
***Prph2***	20,958	Yes	− 1,61	9,62E-35	− 1,44	3,24E-74
*Rbp3*	9662	Yes	− 0,13	6,09E-01	− 0,27	2,02E-02
***Rho***	19,220	Yes	− 1,49	4,58E-25	− 2,10	3,02E-99
***Rp1l1***	6869	Yes	− 1,20	1,27E-13	− 0,41	3,97E-05
*Rpgrip1*	14,808	–	0,01	9,74E-01	− 0,52	3,49E-07
***Sag***	9708	Yes	− 1,44	7,13E-08	− 1,21	2,45E-29
***Slc24a1***	10,736	Yes	− 0,98	8,89E-07	− 0,65	2,62E-10
***Unc119***	10,843	Yes	− 1,08	1,51E-11	− 1,12	7,07E-30

Bold are downregulated photoreceptor (PR) specific genes annotated to downregulated putative eRNAs; FC: fold change

**Fig. 7:**
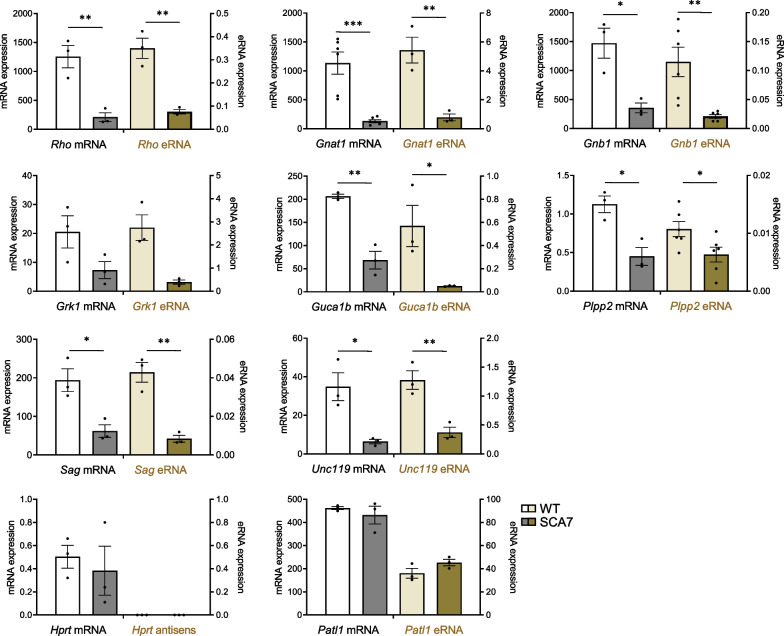
mRNAs and associated putative eRNAs expressed at photoreceptor specific gene loci are downregulated in SCA7 homozygous mice. Graphs showing the RT-qPCR analysis of mRNA and associated putative eRNA levels expressed at photoreceptor gene loci in the retina of SCA7^140Q/140Q^ homozygous mice and WT littermates at 12.5 weeks. All mRNAs and annotated putative eRNAs of photoreceptor genes show downregulation in SCA7 retina compared to WT. The non-deregulated *Patl1* gene and associated non-deregulated non-coding RNA (based on RNA-seq) were used as control. The non-deregulated *Hprt* gene which has no associated lncRNA was used as negative, strand specific control. Data are normalized on *Rplp0* mRNA expression level, expressed as mean ± SEM (n = 3–6) and analyzed using two-tailed Student’s *t*-test; *p < 0.05; **p < 0.01; ***p < 0.001

In SCA7 retina, H3K9ac and H3K27 profiles were reduced at photoreceptor specific gene loci (Fig. 4), suggesting that enhancer functions are progressively inactivated. To further investigate this process, we examined the expression level of putative eRNAs associated with photoreceptor specific genes, and considered this measure as a prospective marker of enhancer activity. Our RNA-seq data actually showed that 15 (*Fam161a*, *Gnat1*, *Gnb1*, *Grk1*, *Guca1b*, *Plpp2*, *Rho*, *Sag, Slc24a1*, *Unc119*, *Prph2*, *Pde6b*, *Cnga1*, *Arl4d* and *Cngb1*) out of 24 photoreceptor specific putative eRNAs had lower level of expression in SCA7 retina than in WT (Table 1). RT-qPCR analysis confirmed the decreased expression level of selected putative eRNAs and associated mRNAs of *Rho*, *Gnat1*, *Gnb1*, *Grk1*, *Guca1b*, *Plpp2*, *Sag* and *Unc119* genes in additional SCA7^140Q/140Q^ retina (Fig. 7), thus correlating with the reduced broadness of H3K9ac profiles. As control, *Patl1* mRNA and its associated putative lncRNA showed similar expression level in WT and SCA7 retina according to our RNA-seq data and RT-qPCR analysis. Interestingly, the expression of putative eRNAs associated to *Rho*, *Gnat1*, *Gnb1*, *Grk1*, *Guca1b*, *Plpp2*, *Sag* and *Unc119* gene loci was also reduced in symptomatic SCA7^140Q/5Q^ retina, but not in pre-symptomatic ones (Additional file 2: Fig. S11). Taken together, the data show that the decrease in putative eRNAs expression parallels the decrease in H3K9ac broadness and the progressive inactivation of photoreceptor identity genes in the SCA7 retina.

## Discussion

Photoreceptor OS specialization is essential for vision and results from a well-defined pattern of gene expression established during differentiation and maintained throughout life to ensure daily renewal of OS in mature photoreceptors [15, 16]. Previous studies have reported that a few dozen of genes involved in phototransduction are downregulated in SCA7 retina [7–11]. In our study, we revealed a broader list of deregulated genes containing several hundreds of highly expressed identity genes that are preferentially downregulated, providing a molecular explanation for the deficit in OS maintenance and photoreceptor function in SCA7 mouse retina. We uncovered that the identity genes most vulnerable to downregulation harbor a specific epigenetic signature, characterized by unusually broad profiles of SAGA-related H3K9 acetylation spanning the entire gene body and low levels of paused RNAPII. We also found that this signature coincides with other features which typically define active cell-type specific superenhancer regions, such as a broad H3K27 acetylation profile, the presence of binding sites for photoreceptor specific transcription factors and the expression of putative eRNAs. Identity genes downregulated in SCA7 retina show altered broad H3K9ac and H3K27ac profiles and decreased expression of putative eRNAs, suggesting that superenhancer functions supporting their high expression are impaired. Overall, our study provides evidence that distinctive acetylation and transcription features underlying high expression of cell-type specific genes are altered in SCA7 retina, resulting in a defect in the maintenance of identity features of mature photoreceptors.

Earlier analyses of gene promoters in SCA7 transgenic mice have led to conflicting results regarding their acetylation defect [18, 19]. In our study, retinal gene promoters from SCA7^140Q/140Q^ mice showed an overall decrease of H3K9ac and other active epigenetic marks, which correlated with a general increase of paused RNAPII, but not with a global change in gene expression. A weak correlation between changes in gene expression and histone modification has been previously reported in several experimental conditions, including in HD mice [75, 76] and could be explained by different mechanisms [77, 78]. For instance, recent studies in yeast have showed that inhibition of SAGA HAT activity causes a general decrease of histone acetylation and mRNA synthesis that is largely compensated by reduced mRNA decay [67, 68]. In the retina of the new SCA7 knock-in mouse model, disruption of gene promoter acetylation could be insufficient to impact gene transcription or could result in decreased transcription that is compensated by reduced RNA decay as in yeast, leading in both cases to maintenance of steady-state mRNA levels of most expressed genes.

Cumulative evidence has shown that in addition to transcription factors epigenomic organization of chromatin at 1D, 2D and 3D levels plays an important role in photoreceptor differentiation and homeostasis [63, 64, 79–81]. Disruption of chromatin topology at TADs has dramatic consequence for gene regulation and causes retinopathy [82]. Aldiri and coll [64] combined H3K27ac and Brd4 ChIP-seq data to identify developmentally-regulated superenhancers located near photoreceptor genes. Our findings showing that identity gene loci of photoreceptors harbor broad H3K9 acetylation concomitant to H3K27ac and are transcribed into putative eRNAs, highlight additional features that would define photoreceptor-specific superenhancers. Moreover, the broad acetylation of H3K9 and H3K27 reported here extends unusually downstream of the TSS along photoreceptor gene bodies and increases progressively during cell differentiation with the expression of photoreceptor identity gene mRNAs and putative eRNAs. The broad H3K9ac in combination with H3K27ac likely facilitates chromatin unfolding for looping interactions between superenhancers and promoters, while lncRNAs transcribed at enhancers may consolidate 3D chromatin organization by mediating recruitment of the mediator complex as previously shown [83, 84]. Additionally, H3K9ac marks in gene bodies and lncRNAs transcribed at enhancers are also implicated in RNAPII pausing reduction; the former by recruiting chromatin readers such AF9 and ENL [85], and the latter by facilitating the release of the negative elongation factor NELF or by activating the positive transcription elongation factor b (P-TEFb) [86, 87]. Whether specific H3K9ac- and lncRNA-based mechanisms promote superenhancers activity and RNAPII pausing release to support elevated transcription of photoreceptor identity genes will merit further experiments.

Photoreceptor identity genes vulnerable to downregulation in the SCA7 retina harbor a broad profile of histone acetylation as well as binding sites for CRX, NRL and other transcription factors involved in photoreceptor cell fate. These transcription factors are likely involved directly or in combination with other factors in recruiting HAT activities to identity gene loci to establish broad histone acetylation [81]. This has in fact been documented for CRX, which binds regulatory elements of photopigment genes prior to HAT components and histone acetylation [88]. Our analysis suggests that a global CRX and NRL dysfunction per se is unlikely in SCA7 retina. Consistent with this, earlier studies have showed that the binding of CRX on its DNA responsive element was not altered over time in SCA7 retina [8, 19]. The propensity of a subset of CRX and NRL target genes to be downregulated could thus be the consequence of their higher sensitivity to loss of HAT activities that acetylate H3K9 and H3K27 along their loci, which in turn leads to disruptions in the topography of superenhancer that drive transcription dynamics. Growing evidence indicates that superenhancers are vulnerable to disruption, which explains the cell-type specific susceptibility in several human diseases [43, 44, 73, 89]. Lipinski and collaborators [29] recently showed that the epigenetic profile of neuronal genes is not self-sustaining and requires the persistence of CBP and P300 HAT activities at the superenhancer level to maintain the identity of excitatory neurons, hence establishing CBP/P300 duo as guardians of the fate of these neurons. Consistent with Lipinski’s results, an earlier study by Henning et al. [80] showed that specific loss of both CBP and P300 in photoreceptors results in a defect in the maintenance of gene expression and cell identity. In both studies, CBP and P300 were individually dispensable for the maintenance of mature neuron, highlighting some level of redundancy in the epigenetic processes implicated in neuron maintenance. Our findings in SCA7 retina further support the view that continuous and diverse HAT activities are essential for maintaining photoreceptor identity by preserving superenhancers acetylation and, consequently, the expression of cell-type specific genes.

SAGA HAT activity in the retina might rely primarily on PCAF, because *Pcaf* is expressed 7–50 times more than *Gcn5* in mouse retina, according to RNA-seq data from this and another study [64]. This could explain why GCN5 depletion did not exacerbate SCA7 transcriptional alterations in the mouse retina [65]. Interestingly, ATXN7 and PCAF can both interact with CBP, suggesting that cooperative acetylation of H3K9 and H3K27 at gene loci may occur [90, 91]. Various in vitro studies showed that mATXN7 reduces HAT activity of SAGA [19, 20] and that it can repress CBP-dependent transcription [92]. Therefore, mATXN7 within SAGA and through interaction with CBP could alter both HAT activities and compromise the maintenance of broad H3K9 and H3K27 acetylation profiles at photoreceptor gene loci. In addition, proteolytic fragments of mATXN7 abnormally accumulate in photoreceptor nuclei and form polyQ aggregates [93, 94] that have been shown to sequester CBP and SAGA subunits [95], suggesting that sequestration may also result in altered HAT activities of SAGA and CBP. Whether alteration of SAGA and CBP HAT activity directly account for the observed acetylation defects remains to be determined, as both SAGA and CBP have additional functions [6, 96], including interaction with and/or acetylation of transcription regulators [97, 98].

Similar to our findings, hypoacetylation of H3K9 and H3K27 was reported at downregulated striatal genes in HD [22, 75, 76]. Notably, downregulation of striatal genes has also been correlated with decreased broad acetylation of H3K27 at putative superenhancers and altered eRNA transcription [22, 47]. Mutant huntingtin in HD has also been shown to alter various HAT activities, either by direct interaction (e.g. CBP, P300 and PCAF [99]) or through sequestration into polyQ aggregates (e.g. CBP [100]). Furthermore, Galvan et al. [101] recently showed that doublecortin like kinase 3 (DCLK3) provides neuroprotection against striatal degeneration in HD, and hypothesized that DLCK3 may play a role in the transcriptional regulation of striatal genes via its interaction with ADA3, a SAGA subunit necessary for HAT activity [4]. Therefore, similar epigenetic alterations of superenhancer-regulated genes in HD striatum and SCA7 retina may point to common pathomechanisms in polyQ disorders. Regarding this possibility, we have previously shown that the global level of H3K9 acetylation is reduced in the cerebellum of SCA7 mice and correlates with the downregulation of 83 Purkinje cell identity genes, which are also altered in the cerebellum of SCA1 and SCA2 mice [11]. Therefore, a genome-wide approach is warranted to determine whether similar epigenetic pathomechanisms underlie Purkinje cells vulnerability in these ataxias.

Our study also revealed that microglial and glial genes involved in immune response process are upregulated as mouse SCA7 retinopathy worsens. The immune response genes were also upregulated in the affected cerebellum of SCA7 mice [11] and this is a well-known shared feature of polyQ diseases [102]. Microglial and glia activation is possibly a secondary event involved in retina remodeling as outer segments progressively retract. In our epigenetic study, the majority of upregulated immune genes did not have detectable H3K9ac peaks in the retina, indicating that the impact of mATXN7 protein on genome-wide H3K9 acetylation and other epigenetic marks could not be covered for low-abundance retinal cell types, such as microglial and glial cells. Upregulated genes did not have significant cluster enrichment, likely because they are expressed in several retinal cell types and their epigenetic profiles were not captured, unlike the profiles of photoreceptor identity genes. Whether mATXN7 has direct effects on epigenetics and gene transcription in microglial cells, glial cells or other low-abundance cell types in the SCA7 retinopathy would need to be investigated using cell type-specific resolution. Previous studies showed that transgenic mice expressing mATXN7 specifically in cerebellar Bergmann glia have reduced expression of *Glast* gene, encoding an important glial transporter involved in glutamate uptake to prevent excitotoxicity in Purkinje cells [103]. However, compared to SCA7 knock-in mice, these transgenic mice developed mild cerebellar pathology. Nevertheless, it would be of great interest to study the epigenetic acetylation states of glial cells and their relevance to non-cell autonomous retinal and cerebellar neurodegeneration.

## Conclusion

Taken together, our genome-wide study of SCA7 retinopathy provides evidence for a predominant downregulation of identity genes of mature neurons, whose cell-type specific high expression is associated with distinct transcriptional and epigenetic signatures. The results also suggest that a critical role of ATXN7, and by extension of SAGA-mediated acetylation, in maintaining cell-type specific gene expression of mature neurons is impaired and accounts for visual impairment in SCA7 mouse retina. Our findings thus refine our view of the mechanisms underlying the loss of neuronal function in SCA7 pathology.

## Supplementary Information

**Additional file 1: Table S1.** List of the primers used in the study. **Table S2.** Photoreceptor specific gene dataset. **Table S3.** Housekeeping gene dataset. **Table S4.** CRX direct gene targets. **Table S5.** NRL direct gene targets. **Table S6.** Genes downregulated in SCA7 (FC < 0.7, p < 0.05). **Table S7.** 71 genes coding for proteins located in cilium and OS structures and downregulated in SCA7. **Table S8.** Genes upregulated in SCA7 (FC > 1.3, p < 0.05). **Table S9.** Occurrence of TF binding motifs in genomic loci associated with cluster 10. **Table S10.** Genomic coordinates of 268 super-enhancers predicted by ROSE. **Table S11.** Genic properties of lncRNAs identified in the retina.

**Additional file 2: Figure S1.** Onset and progression of SCA7 mice retinopathy. **Figure S2.** Specific features of genes and proteins downregulated in SCA7 mouse retina. **Figure S3.** Specific features of genes and proteins upregulated in SCA7 mouse retina. **Figure S4.** Expression of *Crx* and *Nrl* and their target genes in SCA7 retina. **Figure S5.** Enrichment of H3K9ac, H3K27ac, H3K4me1 and RNAPII, densities at expressed gene loci. **Figure S6.** Genome-wide analysis H4K4me1 at gene bodies in SCA7 retina. **Figure S7.** Epigenetic profiles of non-deregulated and upregulated genes. **Figure S8. **Progressive increased broadness of H3K9ac and H3K27ac deposition during retina development correlates with the increased expression of photoreceptor specific genes. **Figure S9.** Identification and validation of putative eRNAs associated to photoreceptor specific gene loci. **Figure S10.** Levels of mRNAs and associated putative eRNAs expressed at photoreceptor specific gene loci increase concomitantly during rod morphogenesis. **Figure S11.** mRNAs and associated putative eRNAs expressed at photoreceptor specific gene loci are downregulated in symptomatic, but not presymptomatic SCA7 retina. Methods of supplementary results.

## Data Availability

The datasets have been submitted to the NCBI Gene Expression Omnibus (GEO) (http://www.ncbi.nlm.nih.gov/geo/) under accession No. GSE181325. Other data that support the findings of this study are available from the corresponding authors on reasonable request.

## Abbreviations

ACT: Actin
ATXN7: Ataxin-7
mATXN7: Mutant ATXN7
ChIP-seq: Chromatin immunoprecipitation followed by high throughput sequencing
Crx: Cone-rod homeobox protein
DCLK3: Doublecortin like kinase 3
eRNA: Enhancer-related lncRNA
HAT: Histone acetyl transferase
HD: Huntington’s disease
H3K9ac: Histone H3 with acetylated lysine 9
H3K27ac: Histone H3 with acetylated lysine 27
H3K4me1: Histone H3 with methylated lysine 4
lncRNA: Long non coding RNA
NRL: Neural retina leucine zipper protein
OS: Outer segment
PI: Pausing index
polyQ: Polyglutamine
RNAPII: RNA polymerase II
SAGA: Spt-Ada-Gcn5-Acetyltransferase
SE: Superenhancer
TAD: Topologically associated domain
TSS: Transcription start site
TTS: Transcription termination site
TUB: Tubulin
