# Supplementary material for: Polyglutamine-expanded ATXN7 alters a specific epigenetic signature underlying photoreceptor identity gene expression in SCA7 mouse retinopathy
Source: J Biomed Sci. 2022 Dec 20;29:107. doi: 10.1186/s12929-022-00892-1 (PMC9768914; doi:10.1186/s12929-022-00892-1)
Supplement: Supplementary file 2 — Additional file 2: Figure S1. Onset and progression of SCA7 mice retinopathy. Figure S2. Specific features of genes and proteins downregulated in SCA7 mouse retina. Figure S3. Specific features of genes and proteins upregulated in SCA7 mouse retina. Figure S4. Expression of Crx and Nrl and their target genes in SCA7 retina. Figure S5. Enrichment of H3K9ac, H3K27ac, H3K4me1 and RNAPII, densities at expressed gene loci. Figure S6. Genome-wide analysis H4K4me1 at gene bodies in SCA7 retina. Figure S7. Epigenetic profiles of non-deregulated and upregulated genes. Figure S8. Progressive increased broadness of H3K9ac and H3K27ac deposition during retina development correlates with the increased expression of photoreceptor specific genes. Figure S9. Identification and validation of putative eRNAs associated to photoreceptor specific gene loci. Figure S10. Levels of mRNAs and associated putative eRNAs expressed at photoreceptor specific gene loci increase concomitantly during rod morphogenesis. Figure S11. mRNAs and associated putative eRNAs expressed at photoreceptor specific gene loci are downregulated in symptomatic, but not presymptomatic SCA7 retina. Methods of supplementary results. [file 12929_2022_892_MOESM2_ESM.pdf]

Supplementary figures

Figure S1. Phenotypic features of SCA7 mice retinopathy.

A) Summary of the SCA7$^{140Q/5Q}$ mouse retinopathy. From 10 weeks of age, the retina presents a progressive reduction of the electroretinography (ERG) activity and a significant decrease of photopigment mRNAs levels (cone opsins (*Opn1mw* and *Opn1sw*) and rhodopsin (*Rho*)). At 20-24 weeks, the retina shows important shortening (> 50%) of the outer segment (OS) layer, while other retinal layers show only mild alterations at later stages. (*Percentage of photopic activity measured at 1 c.d.sec and flicker activity at 5Hz in SCA7$^{140Q/5Q}$ retina, compared to activity (100%) of age-matched WT retina. **Percentage of mRNA levels measured in SCA7$^{140Q/5Q}$ retina, compared to levels (100%) in age-matched WT retina. n.s.d.: not significantly different than the level in age-matched WT mice).

B) RT-qPCR analysis showing the comparable reduced expression of *Rho* and *Opn1sw* in the retina of 12.5-week-old SCA7$^{140Q/140Q}$ homozygous (Hmz) and 22-week-old SCA7$^{140Q/5Q}$ heterozygous (Htz) mice. Data (mean ±SEM (*n*= 3-6 mice/genotype)) are normalized to *Hprt* levels and presented relative to wild type (WT) mRNA levels.

C) Electroretinograph activities of the retina of SCA7$^{140Q/140Q}$ mice. The absence of scotopic (left) and photopic (right) responses to all tested flash intensities indicates that both rod and cone functions are severely impaired at 12.5 week-old. Scale bars : 50 ms and 250 µV in scotopic conditions, 50 ms and 50 µV in photopic conditions.

D) Histology of the retina of SCA7$^{140Q/140Q}$ mice. Histologic sections and electron micrographs show the shortening of outer segment (OS) layer at 5 weeks of age and an almost complete disappearance of OS at 12.5 weeks, compared to WT retina; yellow asterisks in electron micrograph : OS remnants; RPE, retinal pigmented epithelium; IS, inner segment; ONL, outer nuclear layer; INL, inner nuclear layer; IPL, inner plexiform layer; GCL, ganglion cell layer.

Figure S2. **Specific features of genes and proteins downregulated in SCA7 mouse retina.**

A) Venn diagram showing the overlap between 907 downregulated genes in SCA7 retina and genes reported to be expressed in purified rod (13553 genes) and cone (12951 genes) photoreceptors from WT retina (ref. 63). 750 out of 907 are genes expressed in photoreceptors, and most of them likely in rods as they constitute approximatively 80% of retinal cells, while cones represent only 2%.

B) Genetic network showing partnerships and functional interactions between proteins encoded by 71 downregulated genes involved in ciliary and OS functions ($p < 1.0 \times 10^{-16}$) (STRING v.11). Light blue line represents the protein relationships based on curated databases; pink represents experimentally determined data; light green represents text mining; black represents co-expression. The assigned

GO/pathway are indicated: yellow – detection of light stimulus (BP); red – photoreceptor outer segment (OS); green – cilium (CC); blue – phototransduction (KEGG pathway).

C) Western blot analysis showing the progressive reduced expression of Rhodopsin (RHO), cone Arrestin (ARR3) and Recoverin (RCVRN) proteins in SCA7$^{140Q/140Q}$ retina (Hmz) , compared to aged-matched WT. Actin (ACTB) is used as a marker of protein amount. The fold change HMZ/WT of *Arr3*, *Rho* and *Rcvrn* at the RNA level for 12.5-week animals was, respectively, 0.11, 0.23 and 0.59. MW, molecular weight; wk, week.

Figure S3. **Specific features of genes and proteins upregulated in SCA7 mouse retina.**

A) Genetic network showing partnerships and functional interactions between proteins associated with immune response functions. The network (p value: 4.6 x 10^{-135}) was obtained based on analysis of upregulated genes with David software and visualized using STRING v 11.

B) Western blot analysis showing the activation of glial and microglial responses in the retina of 12.5-week-old SCA7$^{140Q/140Q}$ homozygous (Hmz) mice. Levels of the glial GFAP and microglial IBA1 markers are significantly increased in SCA7 retina compared to age-matched WT. Data are normalized to GAPDH level and expressed as mean ±SEM (n= 5 mice/genotype), and analyzed using two-tailed Student's t-test: *** $p < 0.001$.

C) Immunofluorescence analysis showing the timeline of glial activation in the retina of SCA7$^{140Q/5Q}$ heterozygous (Htz) mice. Upper panel: abnormal accumulation of mutant ATXN7 in nuclei begins at 12 weeks, while GFAP fluorescence is only detected from 27 weeks onward in the projection of Müller glial cells. Lower panel: compared to WT, the immunostaining signal of cone opsin (Opn1mw) has already decreased at 12 weeks and is barely detectable from 19 weeks on. Cone opsin reduction happens much before glial activation. GCL, ganglion cell layer; INL, inner nuclear layer; ONL, outer nuclear layer; OS, outer segment.

Figure S4. Expression of *Crx* and *Nrl* and their target genes in SCA7 retina.

A) RT-qPCR analysis showing early downregulation of *Opn1mw* and *Opn1sw* cone genes and *Rhodopsin* (*Rho*) rod gene (upper panel) and later downregulation of *Crx* and *Nrl* genes (lower panel) in the retina of SCA7$^{140Q/5Q}$ heterozygous mice, relative to WT mRNA levels. Data are expressed as mean ± SEM (n= 3-4 mice/genotype/age) and analysed using two-tailed Student's t-test: * $p < 0.05$, ** $p < 0.01$, *** $p < 0.001$.

B) Venn diagram showing the overlap between 1110 CRX and 681 NRL direct transcriptional targets and 748 rod genes downregulated in SCA7$^{140Q/5Q}$ homozygous mice. Values in brackets show the overlap of CRX and NRL targets with the subset of 371 photoreceptor identity genes.

Figure S5. Enrichment of H3K9ac, H3K27ac, H3K4me1 and RNAPII, densities at expressed gene loci.

A) UCSC genome browser tracks depicting ChIP-seq and RNA-seq data at a representative genomic region of WT retina containing expressed and non-expressed genes. Expressed genes (*Tmcc1*, *9530062K07 Rik*, *Zfand4*, *March8*) are characterized by RNA sequencing (RNA-seq) reads on exons and high peak signals of RNAPII, H3K9ac and H3K27ac at their promoter. Non-expressed genes (*Alox5*, *Olfr211*) are devoid of RNA-seq reads and have background levels or no signal of RNAPII and histone post-translational modifications.

B) Genome-wide analysis showing the enrichment of ChIP-seq densities at the transcription start site (TSS) regions (H3K9ac, H3K27ac, H3K4me1 and RNAPII) of expressed genes compared to intergenic regions, in WT and SCA7 retinas. Data were analyzed using Mann-Whitney test, *** $p < 0.001$.

C) 76% (12660 out of 16711) of protein coding genes expressed in WT retina have annotated RNAPII peaks and define the expressed genes set for further analysis. The 4051 expressed genes devoid of annotated RNAPII peaks are majorly expressed in retinal cell type populations that are less abundant than rod photoreceptors, and hence have no detectable RNAPII occupancy at their promoter. 4514 RNAPII peaks are majorly annotated to non-protein coding genes or to genes which expression level was below the threshold criteria (see methods).

Figure S6. Genome-wide analysis H4K4me1 at gene bodies in SCA7 retina.

Violin plots showing the reduction of H3K4me1 densities on regions from transcription start site (TSS) to transcription termination site (TTS) of non-deregulated and downregulated genes in SCA7 retina, compared to WT. Data were analyzed using Mann-Whitney test, *** $p < 0.001$.

Figure S7. Epigenetic profiles of non-deregulated and upregulated genes.

A) RNA-seq expression of *Hprt* gene and genome browser tracks depicting RNAPII, H3K9ac and H3K27ac distributions in WT and SCA7 retina.

B) RNA-seq expression of upregulated microglial (*Trem2*, *Aif1*) and glial (*Gfap*) *genes* and their respective genome browser tracks depicting RNAPII, H3K9ac and H3K27ac distributions in WT and SCA7 retina.

Figure S8. Progressive increased broadness of H3K9ac and H3K27ac deposition during retina development correlates with the increased expression of photoreceptor specific genes.

Genome browser tracks depicting H3K27ac and H3K9ac densities distribution on photoreceptor specific gene loci during retinal development from E14.5 to P21. ChIP-seq and RNA-seq data are from (ref. 64). Raw ChIP-seq data were reannotated to mm10 before use in this study.

Figure S9. Identification and validation of putative eRNAs associated to photoreceptor specific gene loci.

A) Workflow to identify putative enhancer RNAs (eRNAs). RNA-seq and H3K27ac and H3K9ac ChIP-seq data analysis from WT retina were sequentially integrated. Signals within genic regions (the same transcription direction), defined as the interval starting 3 kb upstream of the TSS and ending 10 kb downstream of the TTS and split-mapped reads were removed. The 4301 putative long non-coding RNAs (lncRNAs) obtained were further filtered for the presence of enhancer regions (50% sequence coverage) using H3K27ac ChIP-seq data, resulting in 683 putative eRNA loci. Among these, 282 overlapped with H3K9ac peaks (50% sequence coverage). 24 H3K27ac/H3K9ac-associated putative eRNA loci were annotated to 24 photoreceptor (PR) specific genes.

B) UCSC view depicting the RNA-seq reads of *Rho* and *Gnat1* mouse loci corresponding to their mRNA and putative eRNA.

C) Validation of the expression of putative eRNAs associated with *Rho*, *Gnat1*, *Gnb1*, *Grk1*, *Guca1b*, *Plpp2*, *Sag* and *Unc119* gene loci in WT retina. RT-qPCR analysis of mRNAs and associated putative eRNAs were performed on retina (R), photoreceptor-enriched (PR) and cerebellar (CB) samples of WT mice. Data are normalized to *Hprt* expression level and expressed as mean ±SEM (n= 3-4 mice/genotype).

Figure S10. Levels of mRNAs and associated putative eRNAs expressed at photoreceptor specific gene loci increase concomitantly during rod morphogenesis.

Graphs showing the RT-qPCR analysis of mRNA and putative eRNA levels expressed at *Rho*, *Gnat1*, *Gnb1*, *Grk1*, *Guca1b*, *Plpp2*, *Sag* and *Unc119* gene loci in WT retina at postnatal days P5, P10 and P21. Data are normalized to *Hprt* mRNA level, expressed as mean ±SEM (n= 3 mice/stage/genotype) and analyzed using two-tailed Student's t-test; * $p < 0.05$, ** $p < 0.01$, *** $p < 0.001$.

Figure S11. mRNAs and associated putative eRNAs expressed at photoreceptor specific gene loci are downregulated in symptomatic, but not presymptomatic SCA7 retina.

Graphs showing the RT-qPCR analysis of mRNA and putative eRNA levels expressed at photoreceptor gene loci in the retina of WT and SCA7$^{140Q/5Q}$ heterozygous mice at 5 and 24 weeks, respectively corresponding to presymptomatic and symptomatic stages in SCA7 retina. The mRNA and annotated putative eRNA of photoreceptor specific genes *Rho*, *Gnat1*, *Grk1*, *Guca1b*, *Plpp2*, *Sag* and *Unc119* show downregulation in symptomatic SCA7 retina, but not in presymptomatic ones, as compared to WT littermates. *Gnb1* mRNA and putative eRNA show concomitant downregulation already at 5 weeks.

The non-deregulated *Patl1* gene and associated non-deregulated putative lncRNA (based of RNA-seq) were used as control. Data are normalized on *Hprt* mRNA expression level, expressed as mean ±SEM (n= 3 mice/genotype) and analyzed using two-tailed Student's *t*-test; * $p < 0.05$; ** $p < 0.01$; *** $p < 0.001$.

Figure S1

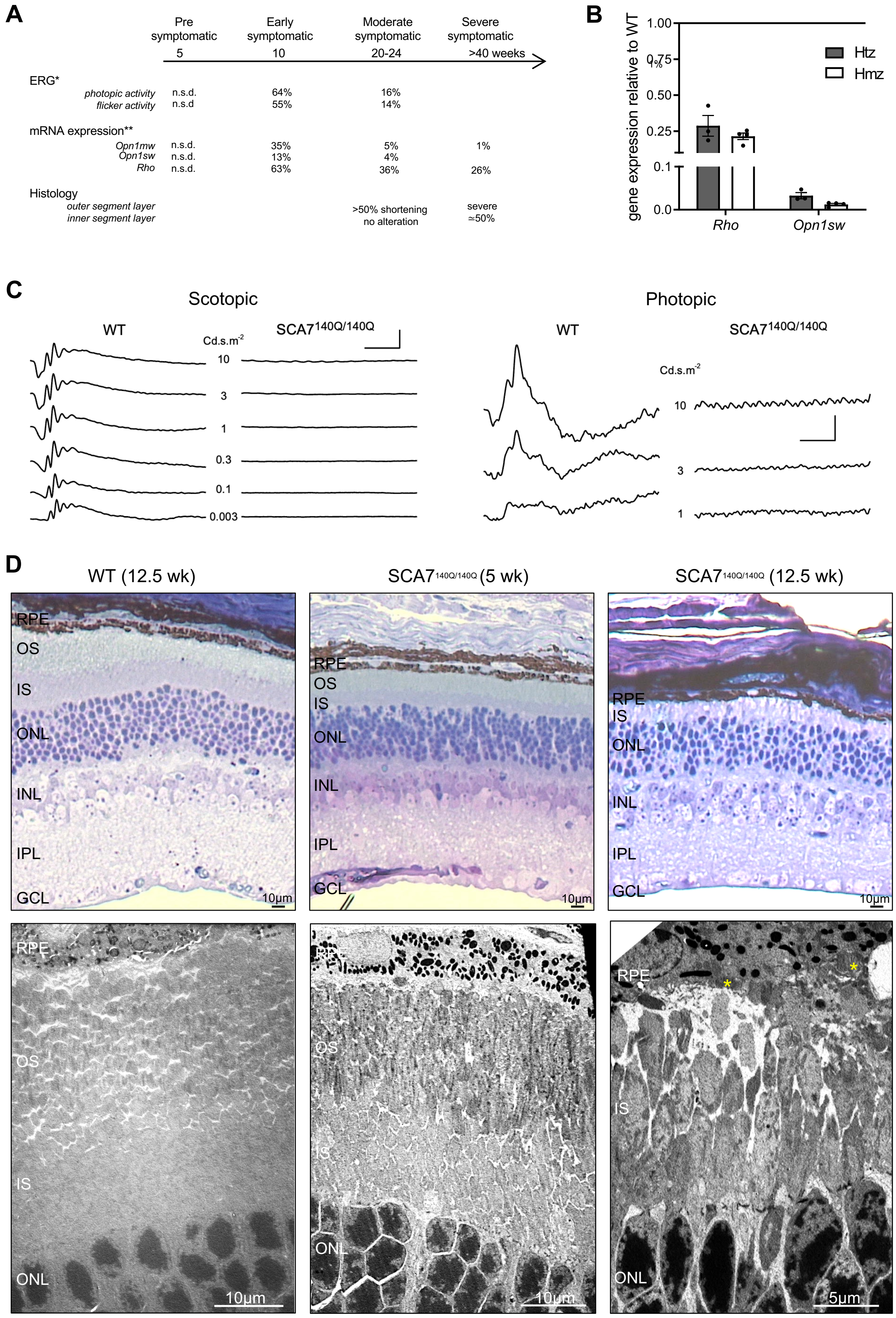
A
Pre symptomatic
Early symptomatic
Moderate symptomatic
Severe symptomatic
5
10
20-24
>40 weeks
ERG*
photopic activity n.s.d. 64% 16%
flicker activity n.s.d 55% 14%
mRNA expression**
Opn1mw n.s.d. 35% 5% 1%
Opn1sw n.s.d. 13% 4%
Rho n.s.d. 63% 36% 26%
Histology
outer segment layer >50% shortening severe
inner segment layer no alteration ≈50%
B
gene expression relative to WT
1.00
0.75
0.50
0.25
0.1
0.0
Htz
Hmz
Rho
Opn1sw
C
Scotopic
Photopic
WT
SCA7140Q/140Q
Cd.s.m-2
10
3
1
0.3
0.1
0.003
D
WT (12.5 wk)
SCA7140Q/140Q (5 wk)
SCA7140Q/140Q (12.5 wk)
RPE
OS
IS
ONL
INL
IPL
GCL
10µm
5µm

Figure S2

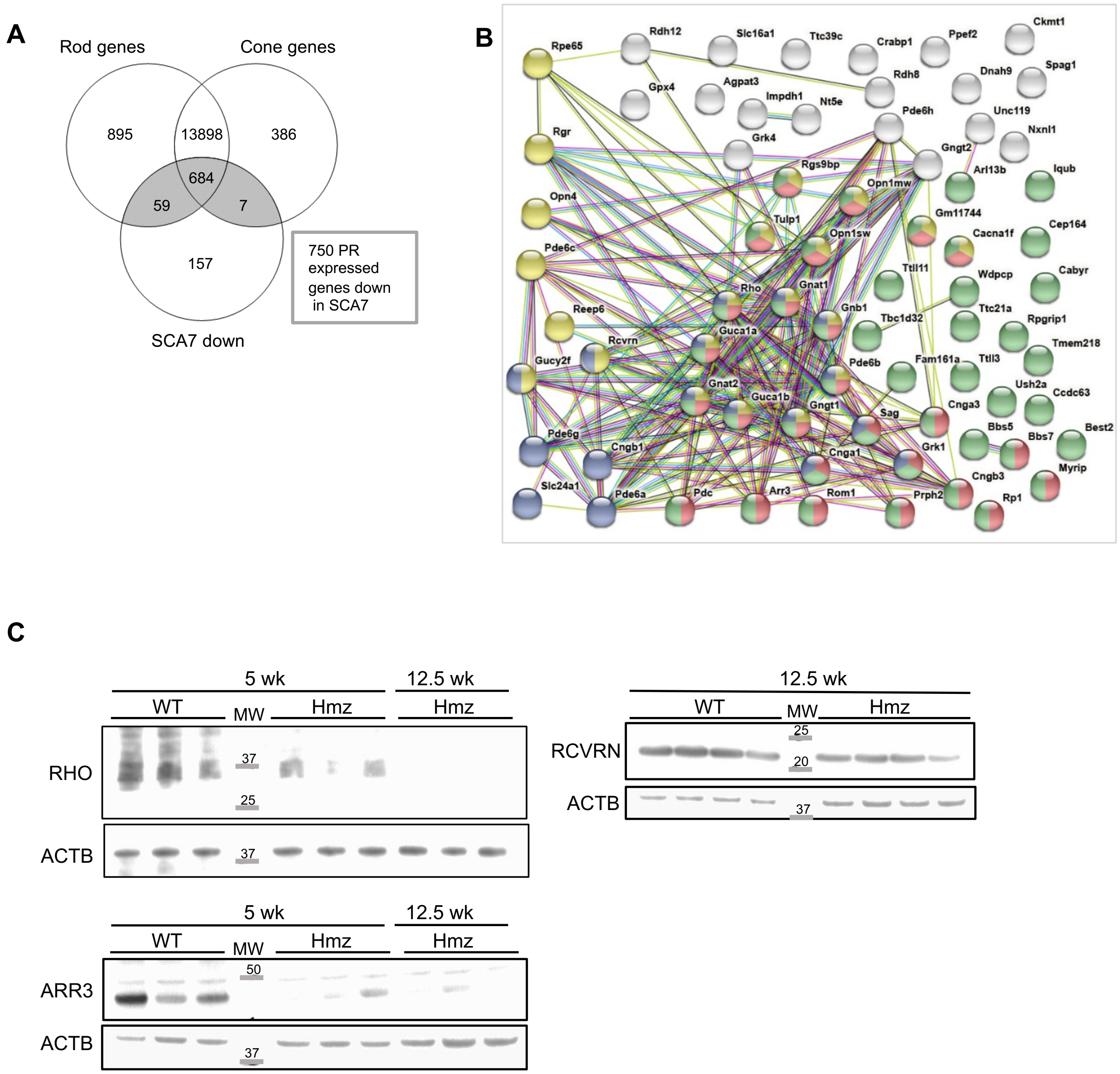
A
Rod genes
Cone genes
895
13898
386
684
59
7
157
SCA7 down
750 PR expressed genes down in SCA7
B
Rpe65
Rdh12
Slc16a1
Ttc39c
Crabp1
Ppef2
Ckmt1
Rdh8
Dnah9
Spag1
Gpx4
Agpat3
Impdh1
Nt5e
Pde6h
Unc119
Nxnl1
Rgr
Grk4
Gngt2
Arl13b
Iqub
Rgs9bp
Opn1mw
Opn4
Tulp1
Gm11744
Opn1sw
Cacna1f
Cep164
Pde6c
Ttll11
Wdpcp
Cabyr
Rho
Gnat1
Gnb1
Reep6
Tbc1d32
Ttc21a
Rpgrip1
Guca1a
Rcvrn
Tmem218
Gucy2f
Pde6b
Fam161a
Ttll3
Gnat2
Ush2a
Guca1b
Gngt1
Cnga3
Ccdc63
Sag
Pde6g
Bbs5
Best2
Cngb1
Grk1
Bbs7
Cnga1
Cngb3
Myrip
Slc24a1
Pde6a
Pdc
Arr3
Rom1
Prph2
Rp1
C
5 wk
12.5 wk
WT
MW
Hmz
RHO
37
25
ACTB
37
RCVRN
25
20
ACTB
37
ARR3
50
ACTB
37

Figure S3

A

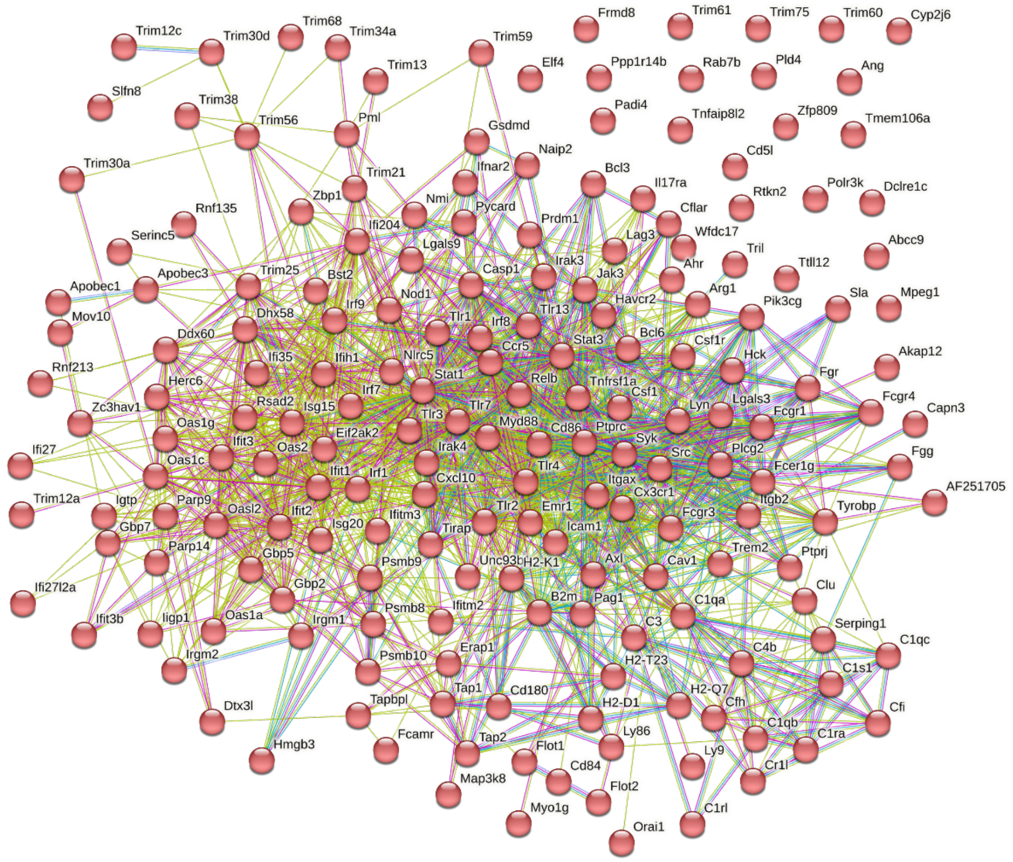

B

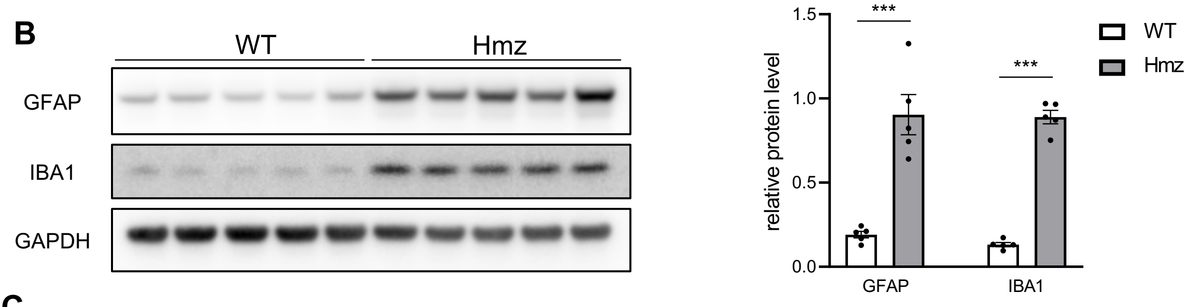

C

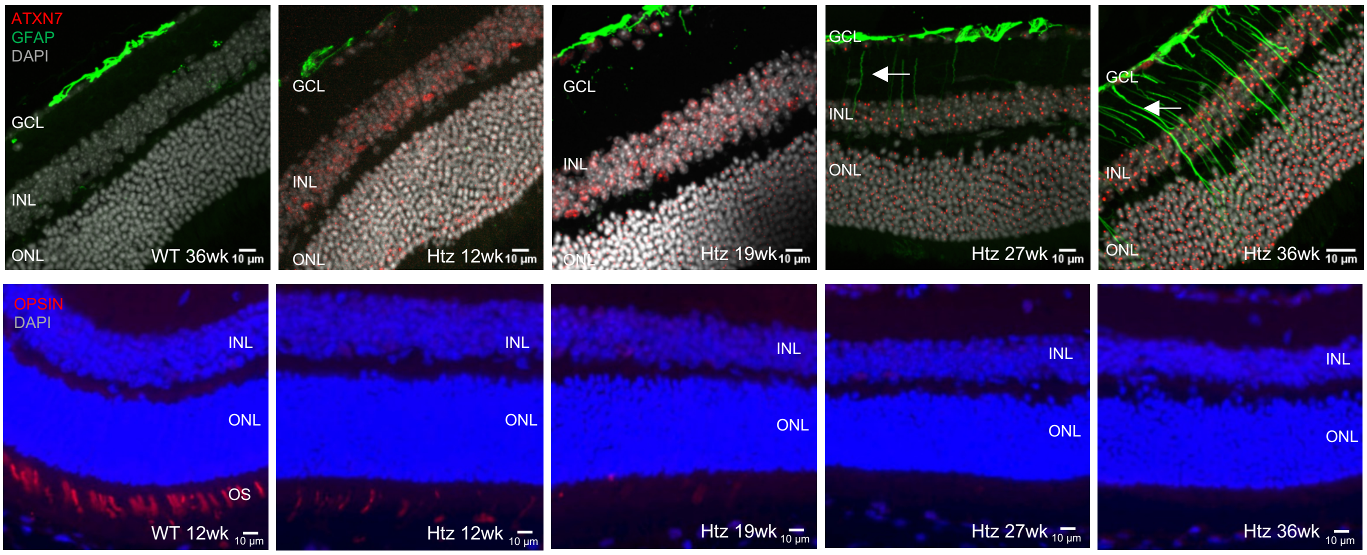

Figure S4

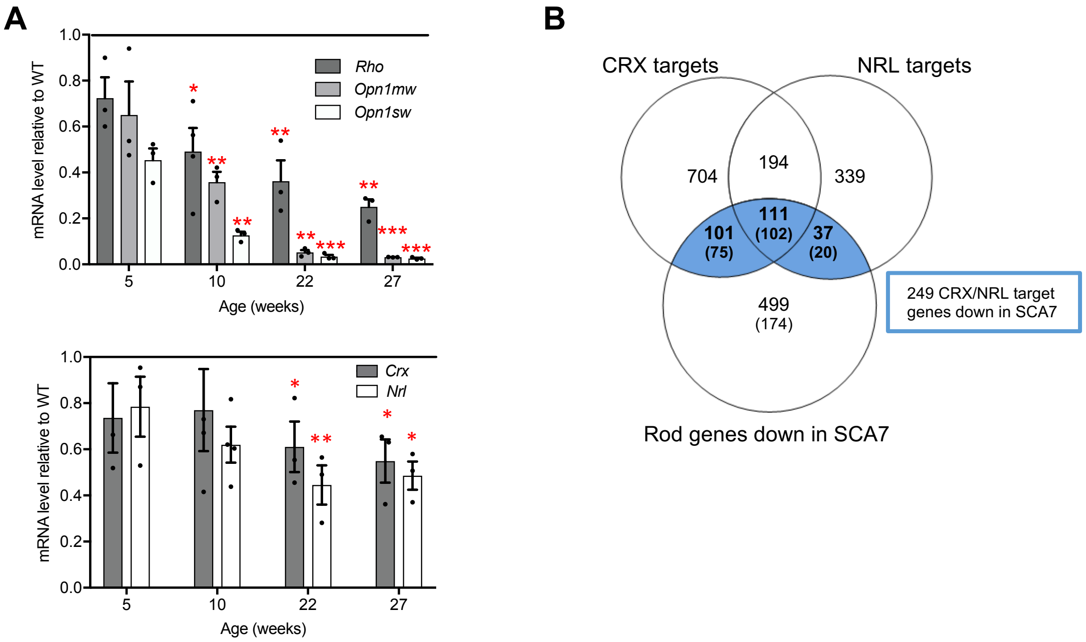
A
1.0
0.8
0.6
0.4
0.2
0.0
mRNA level relative to WT
Rho
Opn1mw
Opn1sw
*
**
**
**
**
**
***
**
***
***
5
10
22
27
Age (weeks)
1.0
0.8
0.6
0.4
0.2
0.0
mRNA level relative to WT
Crx
Nrl
*
**
*
*
5
10
22
27
Age (weeks)
B
CRX targets
NRL targets
704
194
339
101
(75)
111
(102)
37
(20)
499
(174)
Rod genes down in SCA7
249 CRX/NRL target
genes down in SCA7

Figure S5

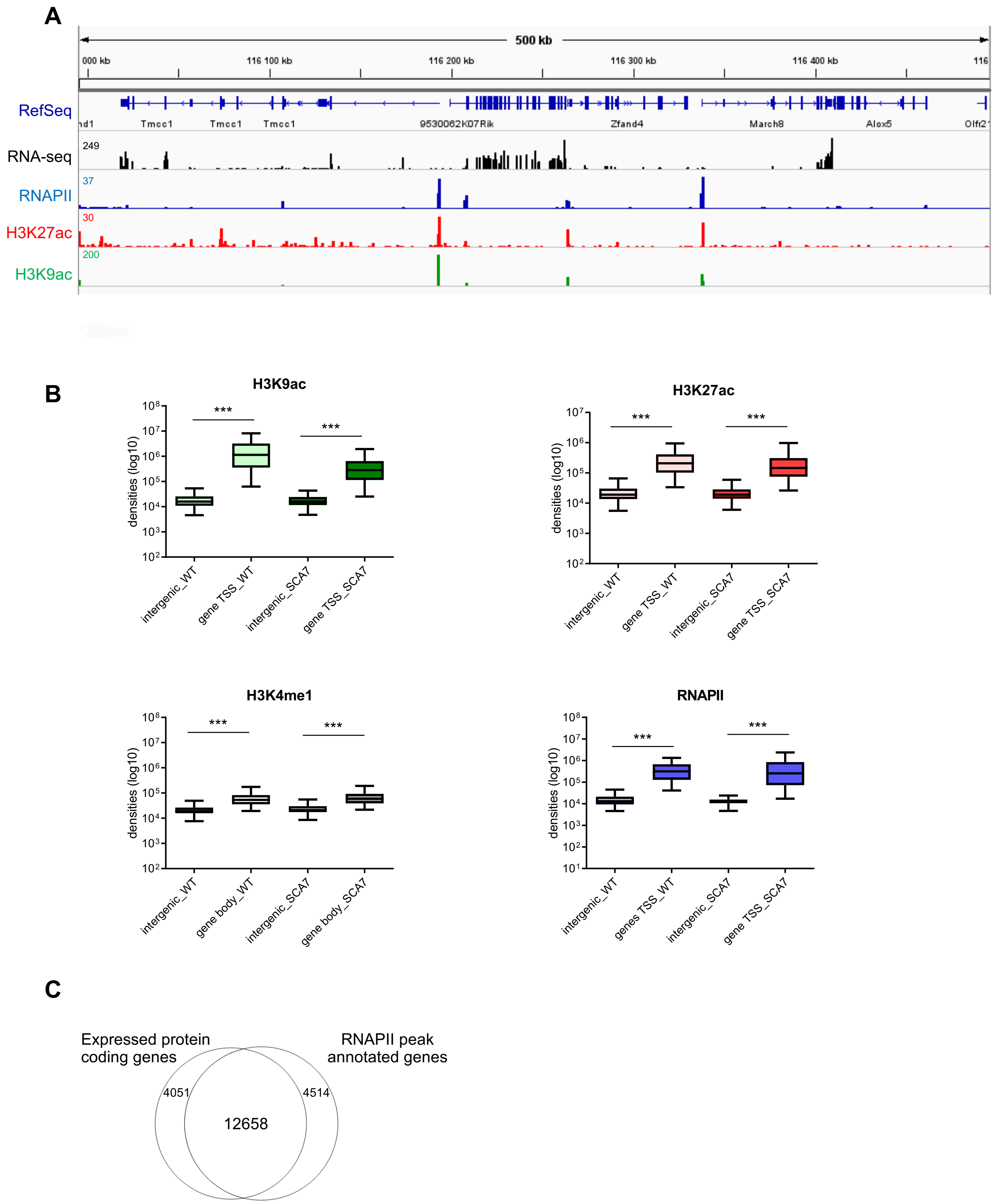
A
500 kb
000 kb
116 100 kb
116 200 kb
116 300 kb
116 400 kb
116
RefSeq
RNA-seq
RNAPII
H3K27ac
H3K9ac
249
37
30
200
Tmcc1
Tmcc1
Tmcc1
9530062K07Rik
Zfand4
March8
Alox5
B
H3K9ac
H3K27ac
H3K4me1
RNAPII
densities (log10)
***
intergenic_WT
gene TSS_WT
intergenic_SCA7
gene TSS_SCA7
gene body_WT
gene body_SCA7
genes TSS_WT
C
Expressed protein coding genes
RNAPII peak annotated genes
4051
12658
4514

Figure S6

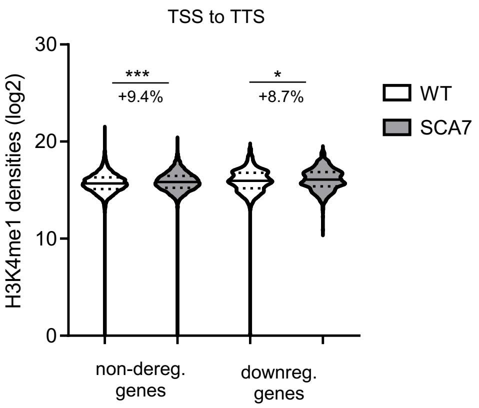
TSS to TTS
H3K4me1 densities (log2)
30
20
10
0
***
+9.4%
*
+8.7%
WT
SCA7
non-dereg.
genes
downreg.
genes

Figure S7

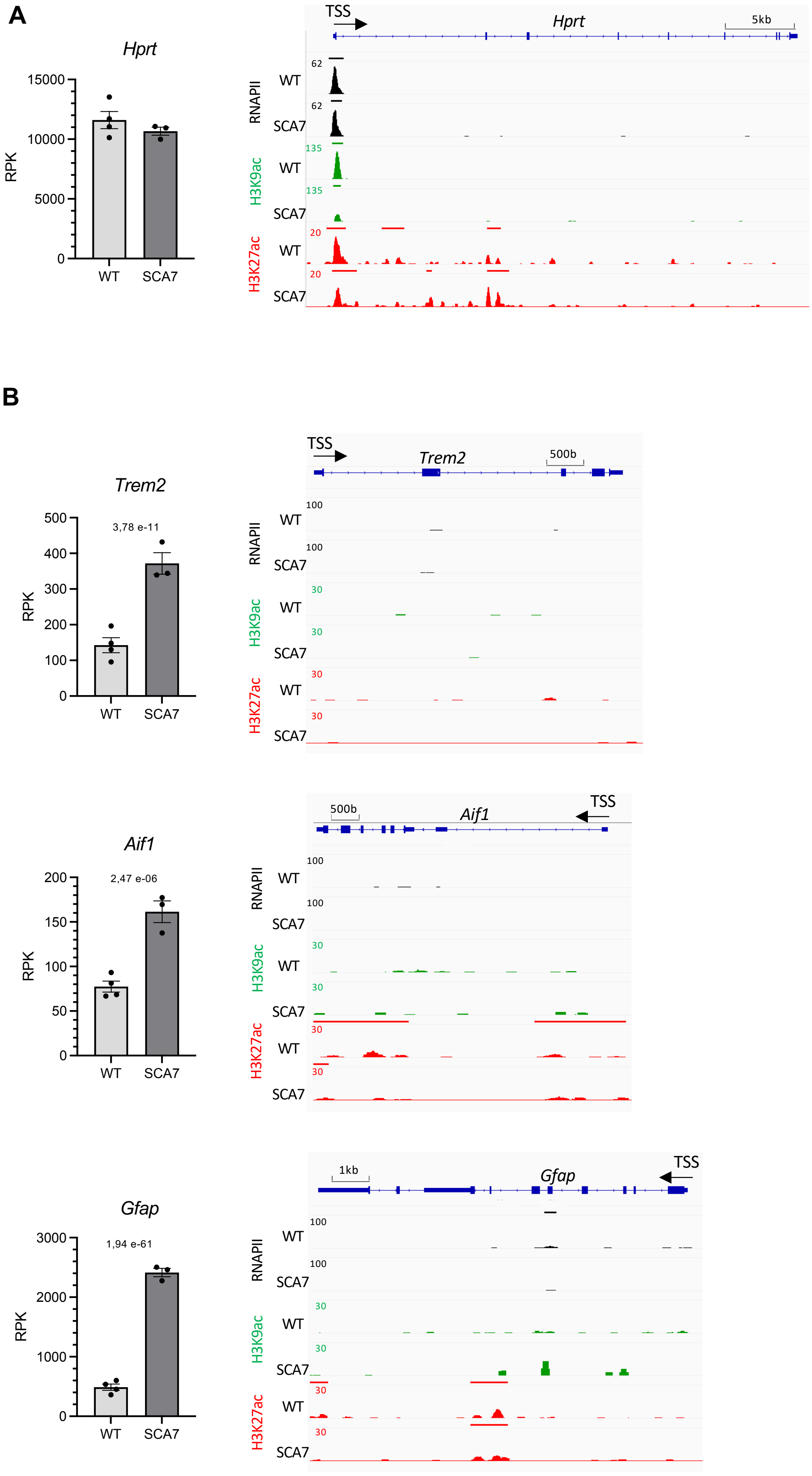
A
Hprt
15000
10000
5000
0
RPK
WT
SCA7
TSS
Hprt
5kb
RNAPII
H3K9ac
H3K27ac
62
135
20
B
Trem2
500
400
300
200
100
0
3,78 e-11
500b
100
30
Aif1
200
150
100
50
2,47 e-06
Gfap
3000
2000
1000
1,94 e-61
1kb

Figure S8

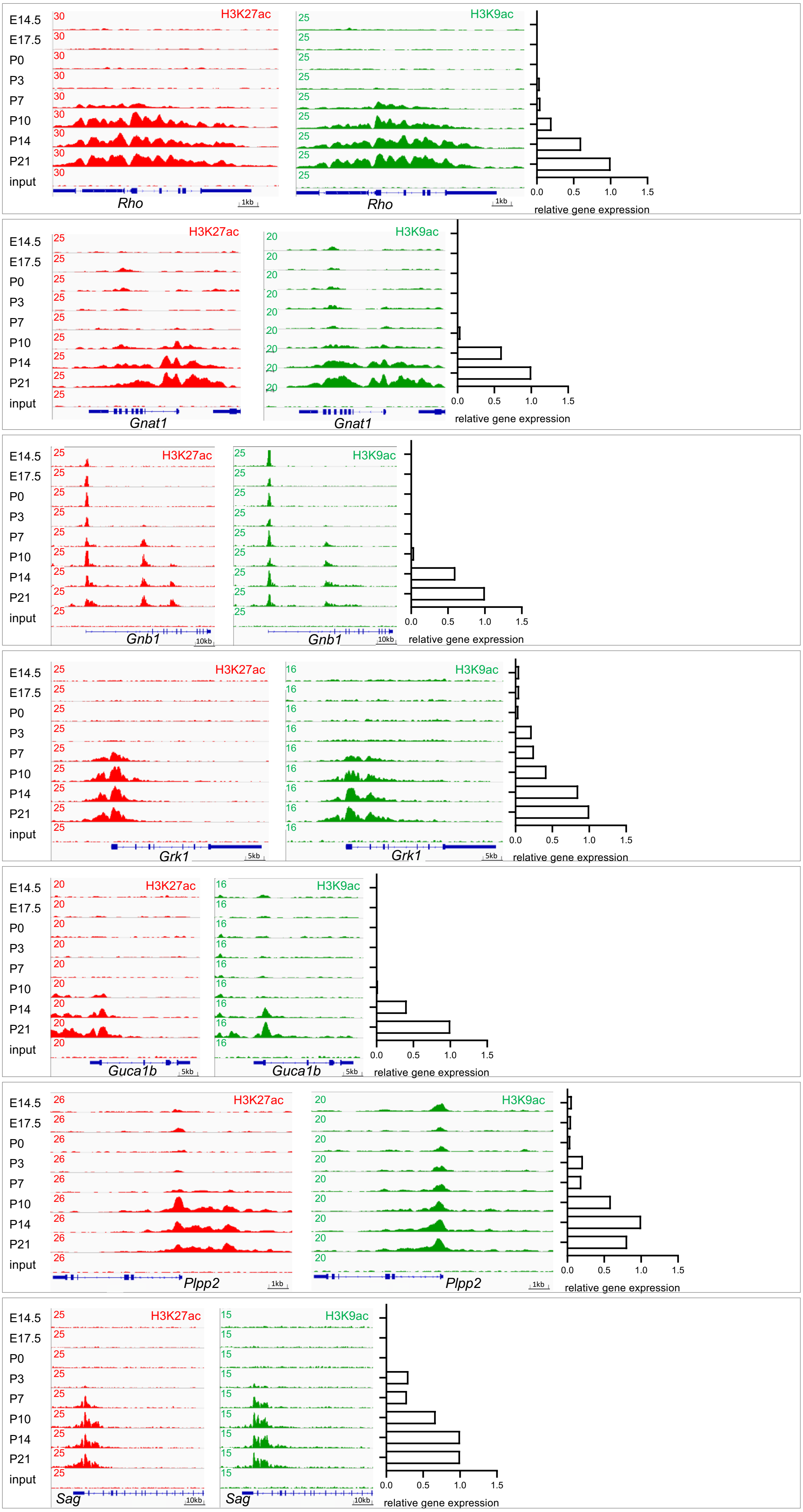

E14.5
E17.5
P0
P3
P7
P10
P14
P21
input
H3K27ac
H3K9ac
Rho
Gnat1
Gnb1
Grk1
Guca1b
Plpp2
Sag
1kb
5kb
10kb
0.0
0.5
1.0
1.5
relative gene expression

Figure S8

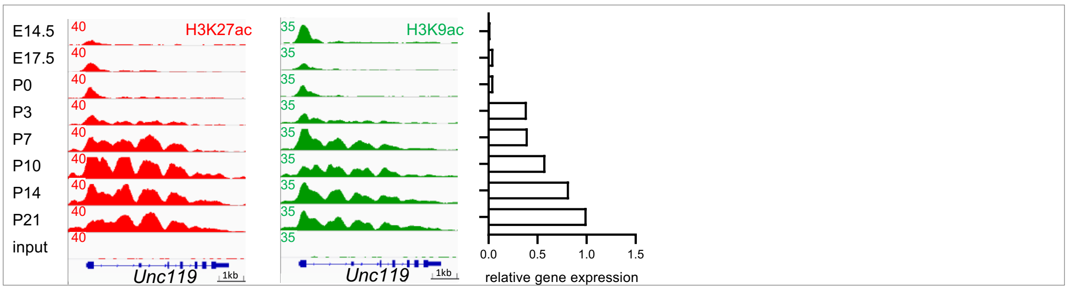

E14.5
E17.5
P0
P3
P7
P10
P14
P21
input
H3K27ac
H3K9ac
40
35
Unc119
1kb
0.0
0.5
1.0
1.5
relative gene expression

Figure S9

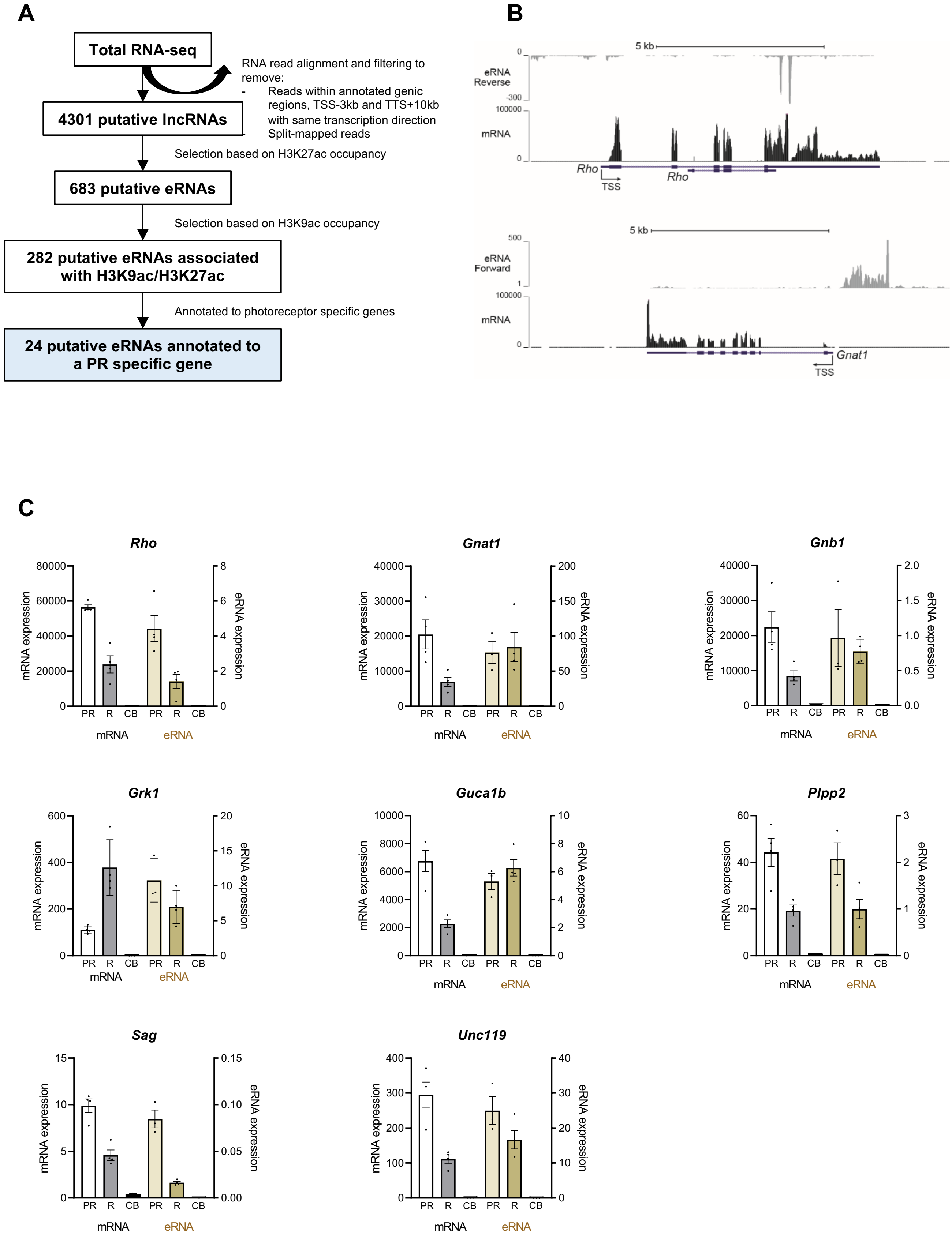
A
Total RNA-seq
RNA read alignment and filtering to remove:
- Reads within annotated genic regions, TSS-3kb and TTS+10kb with same transcription direction
Split-mapped reads
4301 putative lncRNAs
Selection based on H3K27ac occupancy
683 putative eRNAs
Selection based on H3K9ac occupancy
282 putative eRNAs associated with H3K9ac/H3K27ac
Annotated to photoreceptor specific genes
24 putative eRNAs annotated to a PR specific gene
B
5 kb
eRNA Reverse
mRNA
Rho
TSS
Rho
eRNA Forward
mRNA
Gnat1
TSS
C
Rho
Gnat1
Gnb1
Grk1
Guca1b
Plpp2
Sag
Unc119
mRNA expression
eRNA expression
PR R CB PR R CB
mRNA
eRNA

Figure S10

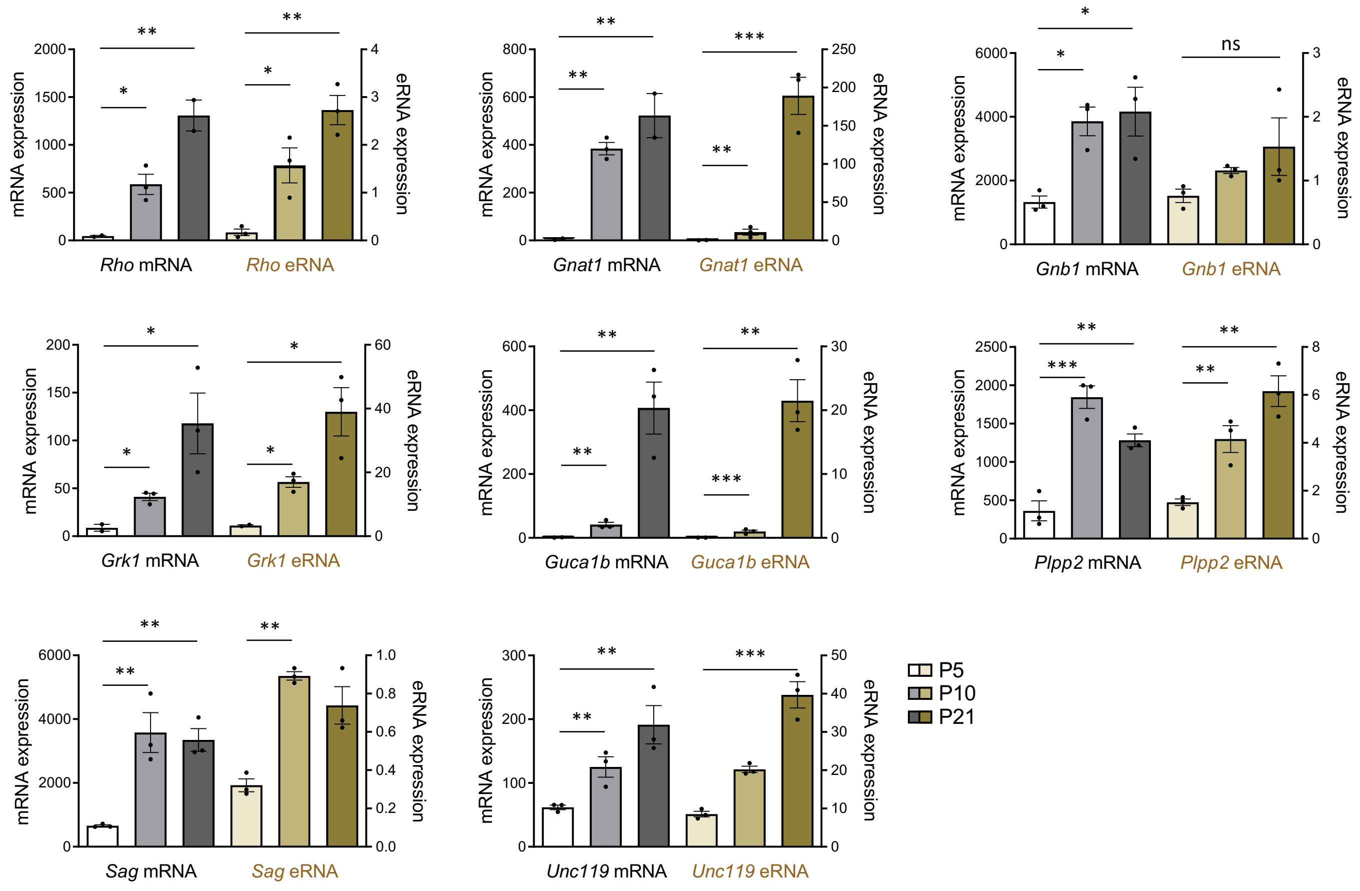

mRNA expression
eRNA expression
Rho mRNA
Rho eRNA
Gnat1 mRNA
Gnat1 eRNA
Gnb1 mRNA
Gnb1 eRNA
Grk1 mRNA
Grk1 eRNA
Guca1b mRNA
Guca1b eRNA
Plpp2 mRNA
Plpp2 eRNA
Sag mRNA
Sag eRNA
Unc119 mRNA
Unc119 eRNA
ns
P5
P10
P21

Figure S11

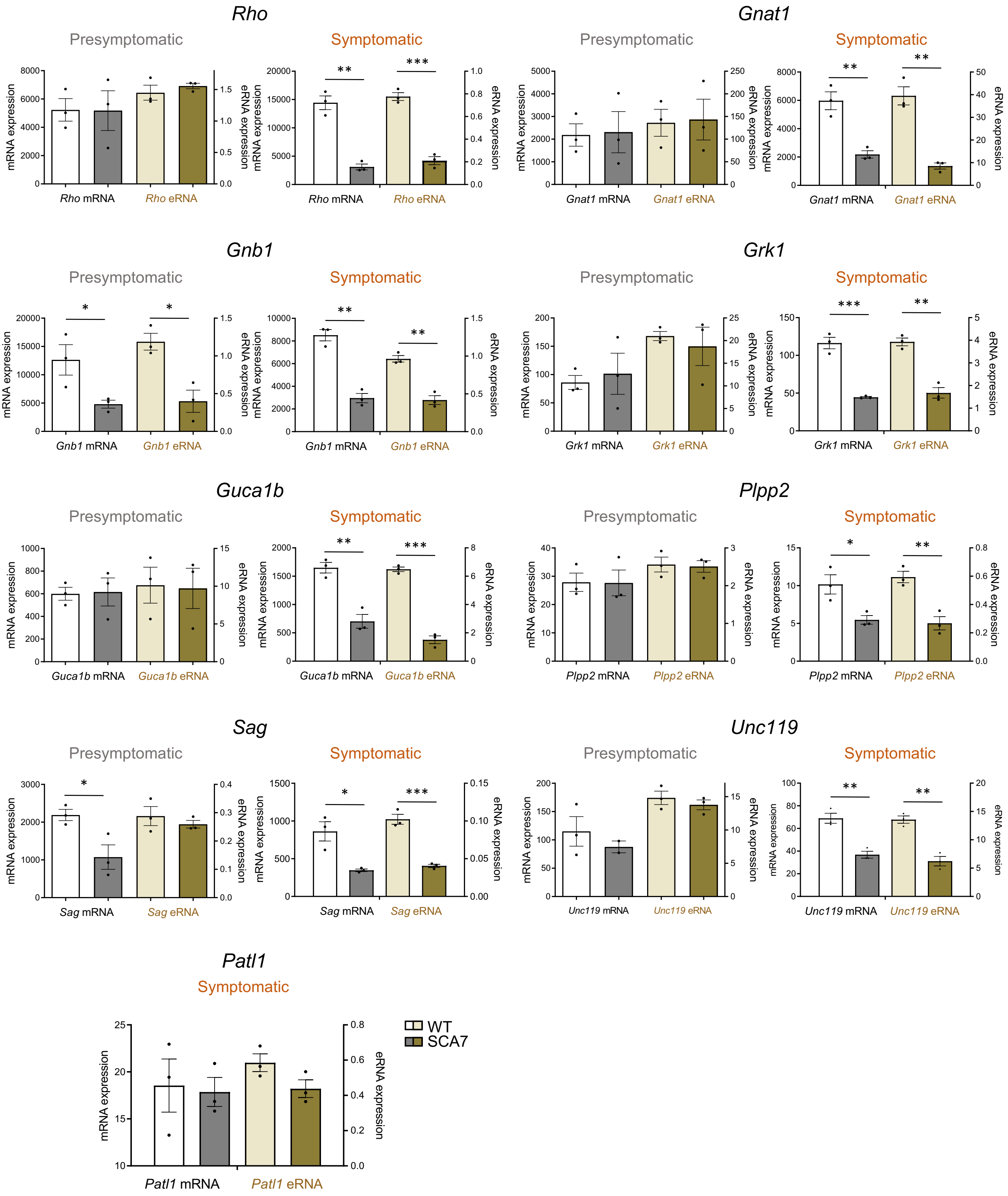

Rho
Presymptomatic
Symptomatic
mRNA expression
eRNA expression
Rho mRNA
Rho eRNA
Gnat1
Gnat1 mRNA
Gnat1 eRNA
Gnb1
Gnb1 mRNA
Gnb1 eRNA
Grk1
Grk1 mRNA
Grk1 eRNA
Guca1b
Guca1b mRNA
Guca1b eRNA
Plpp2
Plpp2 mRNA
Plpp2 eRNA
Sag
Sag mRNA
Sag eRNA
Unc119
Unc119 mRNA
Unc119 eRNA
Patl1
Patl1 mRNA
Patl1 eRNA
WT
SCA7

Supplementary Methods

Electroretinograph activity.

Electroretinographic recordings were obtained using a RETIport system (Roland Consult, Brandenburg an der Havel, Germany). Pupils were dilated with topical application of 0.5% tropicamide (Ciba Vision Ophthalmics, Blagnac, France). Mice were then anesthetized with an intraperitoneal injection of a mixture of ketamine (100mg/kg) and medetomidine (1.2mg/kg). After 5 minutes, animals were positioned on a warming plate set at 37°C to maintain a constant body temperature. Ground and reference (metal needles), and corneal (thin gold wire with a 2-mm ring end) electrodes were placed accordingly. A carbomere gel (Ocry-gel, Virbac, France) was applied to ensure good electrical contact and to keep the cornea hydrated during the entire procedure. Single-flash recordings were obtained under both dark- (scotopic) and light-adapted (photopic) conditions after mice were placed in the Ganzfeld bowl. In scotopic conditions, single-flash stimuli were presented with increasing intensities, from 3×10^{-3} to 10 Cd.s/m^2. Light adaptation was then performed with a background illumination of 30 Cd/m^2 presented 10 min before recording photopic responses to flashes of 1, 3 and 10 Cd.s/m^2. Data were acquired at 1.28 kHz (scotopic) or 2 kHz (photopic) with a 20-300 Hz bandpass filter.

Histology and electron microscopy analysis.

Eyes were prepared for histology and electron microscopy as in (11). Semi-thin (2mm) sagittal sections were cut with an ultramicrotome (Leica Ultracut UCT), stained with toluidine blue, and histologically analyzed by light microscopy. Ultra-thin (70 nm) sections were cut and contrasted with uranyl acetate and lead citrate and examined at 70 kV with a Morgagni 268D electron microscope. Images were captured digitally by Mega View III camera (Soft Imaging System), and contrast was adjusted for display purposes.

Immunofluorescence.

Mice were transcardially perfused with 1x PBS followed by 4% PFA/1x PBS. Eyecups were next incubated in 4% PFA/1x PBS for 2 h and washed 3 times in 1x PBS. After 24 h incubation in 20% sucrose/1x PBS the eyecups were mounted in Shandon Cryomatrix embedding resin (Thermo FisherScientific) and immediately frozen; 12µm eyecup cryosections (Leica Microsystems, CM 3050S) were collected and stored at -80°C. Cryosections were permeabilized in PBSTx (1x PBS/0.1% Triton X-100) and blocked for 1 h at room temperature with PBSTx /deactivated 7% FCS. Sections were incubated o/n at 4°C with primary antibodies in a blocking buffer using the following dilutions: 1:1000 rabbit anti-ATXN7 (1261, IGBMC) 1:700 mouse anti-GFAP (Sigma Millipore, G3893) or 1:1000 rabbit anti-OPN1mw (Sigma Millipore, AB5405). Secondary goat anti-rabbit, goat anti-mouse (Thermo Fisher Scientific, dilution 1:1000) conjugated with Alexa-555, and -488 fluorophores, respectively, were applied for 1h at room temperature. Nuclei were counterstained with 1µg/ml DAPI (4',6-diamidino-2-phenylindole dihydrochloride). Controls without primary antibodies were performed in parallel. Slides were mounted with Aqua Poly Mount (Polysciences), dried and stored light protected at 4°C. Retina images were acquired on a Spinning disk with Leica DMI 8 inverted microscope equipped with Hamamatsu Orca flash 4.0 Camera 5 connected with MetaMorph software (Molecular Devices).

Western blots.

Retinas were dissected and snap frozen in liquid nitrogen. Whole cell extracts were obtained by lysis and sonication of tissues in 150 µl of buffer containing 50mM Hepes-KOH (pH 7.5), 140mM NaCl, 1mM EDTA, 0.1% Na-deoxycholate, 1% SDS, 1% Triton-X100, 1x Protease Inhibitor Cocktail (Roche), 20mM N-ethylmaleide, 20mM nicotinamide. Protein concentrations were measured using Bradford protein assay. 5-30 µg of total proteins was used for electrophoresis. Samples were loaded on 4-12% Bis-Tris protein gels and ran in NuPAGE MOPS SDS Running Buffer (ThermoFisher). Proteins were transferred to nitrocellulose membranes for 1.5 h at 200 mA. Antibodies were diluted in 1x PBSTM (1xPBS with 0.1% Tween, and 5% non-fat milk). The following primary antibodies were added 2 h at room temperature or overnight at 4°C: anti-H3K9ac 1:2 000 (Abcam ab4441); anti-H3K4me1 (Abcam,

ab8895) 1:2 000; anti-H3K27ac (Abcam, ab4729) 1:5 000; anti-H3 1: 5 000 (Abcam, ab1791); anti-GAPDH 1:10 000 (Merck Millipore, MAB374); anti-TUBULIN 1:2 000 (IGBMC, TUB-2A2); anti-IBA1 1:1 000 (Sobioda, W1W019-19741); anti-GFAP 1:700 (Sigma Millipore, G3893); anti-α ACTIN 1:10 000 (IGBMC, 1ACT-2D7); anti-α ACTIN 1:15 0000 (Sigma Millipore, MAB1501); anti-RHODOPSIN 1:500 (Sigma Millipore, MAB5316); anti-cone ARRESTIN 1:500 (Sigma Millipore, AB15282); anti-RECOVERIN 1:3 000 (Sigma Millipore, AB5585). The following secondary antibodies were added in 1x PBSTM for 1h at room temperature and washed three times in 1x PBST. Peroxidase AffiniPure $F(ab')_2$ Fragment Goat Anti-Rabbit IgG (H+L) (GARPO; 1:10 000, Jackson ImmunoResearch Lab.) and peroxidase AffiniPure Goat Anti-Mouse IgG (H+L) (GAMPO; 1:10 000, Jackson ImmunoResearch Lab.). Labelling was revealed using SuperSignal West Pico PLUS Chemiluminescent kit (ThermoFisher) or ECL2 kit (ThermoFisher). Chemiluminescent signal was detected using the Amersham Imager 600 or Autoradiography. Images were analyzed using Fiji.
